# A Rapid Review Contrasting the Evidence on Avian Influenza A(H5Nx) Clades 2.3.4.4b and 2.3.2.1c in Humans

**DOI:** 10.1111/zph.70006

**Published:** 2025-08-26

**Authors:** Tricia Corrin, Kaitlin M. Young, Mavra Qamar, Kusala Pussegoda, Austyn Baumeister, Nicole Atchessi, Erin Leonard, Lisa A. Waddell

**Affiliations:** ^1^ Public Health Risk Sciences Division, National Microbiology Laboratory Public Health Agency of Canada Guelph Ontario Canada; ^2^ Centre for Emerging and Respiratory Infections and Pandemic Preparedness, Infectious Diseases and Vaccination Program Branch Public Health Agency of Canada Ottawa Ontario Canada; ^3^ Centre for Foodborne, Environmental and Zoonotic Infectious Diseases, Infectious Diseases and Vaccination Program Branch Public Health Agency of Canada Halifax Nova Scotia Canada

**Keywords:** 2.3.2.1c, 2.3.4.4b, a(H5Nx), avian influenza, rapid review

## Abstract

Avian influenza viruses (AIV) circulate in wild and domestic bird populations, posing an on‐going risk for zoonotic transmission and virus adaptation to mammals and humans. The A(H5Nx) clades 2.3.2.1c and 2.3.4.4b currently circulating have caused sporadic infections in humans. A rapid review (RR) was conducted to contrast the evidence on infection from these clades in humans. Adhering to PRISMA guidelines, a protocol was developed a priori. The search was conducted in December 2023 for primary research articles (published and preprint) pertaining to AIV clades 2.3.4.4b or 2.3.2.1c in Scopus, PubMed and EuropePMC. Search verification and a grey literature search were also conducted in January 2024. Full‐text relevance screening was conducted independently by two reviewers. Data extraction and risk of bias (ROB) assessment was conducted by one reviewer and verified by a senior reviewer. Results were reported narratively. Forty articles published between 2014 and 2023 were included in this RR. Studies found no discernible difference in the likely mode of exposure, transmission or the potential of AIV infection in humans between the two clades. Seropositivity, seroconversion and hospitalisation rates were also similar, while intensive care unit admission and fatalities were higher for A(H5Nx) clade 2.3.2.1c. Several markers of mammalian adaptation and mutations associated with increased viral replication, polymerase activity and virulence in mammals and/or mice were found in both clades. Most studies were considered to be at high ROB, while some well‐designed cohort studies were at moderate ROB. This summary can be used to inform what is known about A(H5Nx) in humans for the two clades and suggests that there is on‐going adaptation pressure from circulating AIVs that should be closely monitored. It is important to continue surveillance in birds, mammals and humans, conduct large epidemiological studies and develop mitigation strategies from a One Health perspective.

**Summary**

- Markers of mammalian adaptation and mutations associated with increased viral replication, polymerase activity and virulence in mammals and/or mice were found in both A(H5Nx) clades 2.3.2.1c and 2.3.4.4b.
- There was no discernible difference in the likely mode of exposure, transmission or the potential of AIV infection in humans between the two clades.
- For the sporadic cases that have occurred in humans, disease has ranged in severity from asymptomatic/mild to critical and fatal, particularly for those infected with 2.3.2.1c.

## 1 Introduction

The first recorded outbreak of human cases from an avian influenza virus (AIV), A(H5N1), was in China in 1997, resulting in 18 human cases and six deaths (Centers for Disease Control and Prevention 2023a). Since that time, different clades, lineages and subtypes have emerged, and AIVs are well known for acquiring mutations and reassorting, which may result in unpredictable enhancements to the virus' ability to transmit to and infect birds, mammals and humans. The continued circulation of AIVs among wild and domestic bird populations indicates there is an on‐going risk for zoonotic transmission and virus adaptation to mammals and humans (World Health Organization 2023d). This is a significant challenge for global public health and requires a One Health approach to monitoring circulating AIVs in all species for the timely identification of viruses that may have the potential to cause outbreaks in mammals and/or humans and for the initiation of appropriate response activities.

During the last 27 years, numerous AIV subtypes, lineages and clades have caused 996 human cases of A(H5Nx), over 1500 human cases of A(H7N9), and over 130 cases of other A(HxNx) based on official case reporting (Centers for Disease Control and Prevention 2023b; Public Health Agency of Canada 2023). The focus of this rapid review (RR) will be on two AIV A(H5Nx) clades that are circulating in 2024, clade 2.3.2.1c and clade 2.3.4.4b, which have been circulating in birds since 2009 and 2016, respectively. A(H5Nx) clade 2.3.4.4b has caused a panzootic that has affected an unprecedented range of bird species globally as well as a large number of mammalian species associated with over 300 outbreaks in at least 13 countries as of December 2023, suggesting this clade has wide adaptation to a range of birds and may also have some adaptation to mammals (Centers for Disease Control and Prevention 2023a, 2023b; Pabbaraju et al. 2014). Reported spread of clade 2.3.2.1c to new bird species or mammals has been limited in recent years (Centers for Disease Control and Prevention 2023b). Since January 2022, human cases have also been detected for both clades, including six cases of clade 2.3.2.1c A(H5N1) from Cambodia and 13 cases (1 tentative) of clade 2.3.4.4b A(H5N1) from eight countries and 31 cases of A(H5N6), assumed to be clade 2.3.4.4b, in China, January 2022 to December 2023 (Centers for Disease Control and Prevention 2023b; Public Health Agency of Canada 2023). The differences between the two AIV clades are not well known.

This RR aims to contrast the evidence on AIV A(H5Nx) clade 2.3.2.1c and 2.3.4.4b infection in humans regardless of source to understand the transmissibility, pathogenicity, virulence, epidemiological factors, genetic differences and antigenic differences between these clades.

## 2 Methods

### 2.1 Review Type, Protocol and Team

A RR is a type of evidence synthesis that is tailored to answer an urgent research question on a shorter timeline than would be required to conduct a systematic review. The structure and rigour of the methods are similar to a systematic review; however, an RR simplifies or omits parts of the systematic review methodology to speed up the process while maintaining confidence in the findings (Garritty et al. 2021, 2023; Gartlehner et al. 2023; Klerings et al. 2023; Nussbaumer‐Streit et al. 2023). The only shortcut implemented was the replacement of dual independent data extraction and risk of bias assessment with one person extracting the data and a senior person verifying its accuracy. This is considered to have minimal impact on the review given the tools were pretested by all members of the review team.

A protocol was created a priori to ensure transparency, reproducibility and consistency during all stages of the RR (Data S1). It includes a list of definitions, inclusion/exclusion criteria, search strategy, full‐text screening form and the data characterisation form. The Preferred Reporting Items for Systematic Reviews and Meta‐Analyses (PRISMA) 2020 statement was used to guide the preparation of the manuscript where applicable (Page et al. 2021). A multidisciplinary team with expertise in evidence synthesis, epidemiology, infectious diseases, avian influenza and public health created the protocol and executed the RR.

### 2.2 Review Question and Eligibility Criteria

The objective of this RR was to identify, summarise and contrast evidence on relevant clades that address three research questions:

1. Is there a difference in animal‐to‐human transmissibility between avian influenza clade 2.3.4.4b and 2.3.2.1c viruses?
2. Do the epidemiological characteristics of human cases differ between avian influenza clade 2.3.4.4b and 2.3.2.1c viruses?
3. For avian influenza clade 2.3.4.4b and 2.3.2.1c viruses in humans, are there differences in the prevalence of molecular signatures associated with mammalian adaptation, transmissibility, pathogenicity or virulence?

The following inclusion criteria were applied:

1. Publication date: All
2. Language: English and French
3. Study design: All
4. Country: All
5. Population: Humans
6. Document type: Primary research (authors collected and analysed their own data)
7. Pathogens: Avian Influenza clades 2.3.4.4b and 2.3.2.1c

### 2.3 Search Strategy, Grey Literature and Verification

A comprehensive search strategy was developed by the research team and applied in three bibliographic databases to capture both published and preprint articles: Scopus, PubMed and EuropePMC (Data S1). The search was conducted on December 14, 2023, and had no date or language limits.

Search verification was conducted January 15, 2024, to ensure the database search captured all relevant primary research. The reference lists of four relevant reviews containing information on either clade were hand searched and yielded no new articles (Chauhan et al. 2020; Chauhan and Gordon 2022; Kalonda et al. 2021; Tahmo et al. 2023). A grey literature search was conducted January 15, 2024, to identify recent summary reports and the most recent case and outbreak reports. The websites of the World Health Organization, Food and Agricultural Organization, European Food Safety Authority, the US Center for Disease Control, the Pan American Health Organization and the UK Health Security Agency avian influenza webpages were examined for recent reports. From these organisations, five recent summary reports reference lists (Centers for Disease Control and Prevention 2023b; European Food et al. 2023; Food and Agricultural Organization of the United Nations 2023; Pan American Health Organization and World Health Organization 2023; World Health Organization 2024) were screened to identify relevant citations omitted by the electronic search, and a total of five primary research reports (UK Health Security Agency 2023; World Health Organization 2023a, 2023b, 2023c, 2023e) were added to the RR from the grey literature search.

### 2.4 Review Management

The search results were imported into RefWorks (ProQuest LLC), a citation management software, and duplicates were removed. The unique articles were then imported into the web‐based systematic review software DistillerSR (Version 2023.6.2; Evidence Partners, 2023) where all stages of the RR were conducted, including relevance screening, data extraction and risk of bias (ROB) assessment.

### 2.5 Relevance Screening and Data Extraction

To determine if an article was relevant to the review, the inclusion and exclusion criteria were used to design a full‐text relevance screening form. Full‐text relevance screening was conducted independently by two reviewers. For data extraction, a form was created to extract data on key information from each study such as publication details (e.g., year, language), study details (e.g., study design, sampling location and sampling frame, population characteristics), specific clade of avian influenza and data on key outcomes to answer the research questions. Data extraction was conducted by one reviewer and verified by a senior reviewer. Both the relevance screening and data extraction forms were developed a priori and piloted by all reviewers. Piloting the forms enabled modifications and clarifications to be made based on reviewer feedback to ensure consistency before screening and extraction commenced. All conflicts were resolved by consensus or a third reviewer. The screening and data extraction forms including all definitions are outlined in the protocol (Data S1). A list of excluded studies and the reasons for exclusion are documented (Data S2).

### 2.6 Risk of Bias

The articles in this RR were assessed for their ROB based on their study design by the following tools: Joanna Briggs Institute (JBI) critical appraisal checklist for case reports (Moola et al. 2020) and case series (Munn et al. 2020), JBI critical appraisal checklist for prevalence studies and adapted for surveillance data analyses (Munn et al. 2015), Newcastle‐Ottawa Scale (NOS) for cohort studies (Wells et al. 2021) and the adapted version of the NOS for cross‐sectional studies (Herzog et al. 2013) (Data S1, Appendix 1). A validated ROB tool was not available for the following study designs: biological monitoring, outbreak investigations, in vitro and in silico. Therefore, ROB was not assessed for articles with these study designs. The ROB was conducted by one reviewer and verified by a senior reviewer. All conflicts were resolved by consensus or a third reviewer; results are available in Data S2.

### 2.7 Data Analysis and Reporting

Data cleaning, categorisation, descriptive analysis and narrative summarisation were conducted in Microsoft Excel for Microsoft 365 MSO (Version 2307 Build 16.0.16626.20198). The complete dataset is available in Data S2.

## 3 Results

**FIGURE 1:**
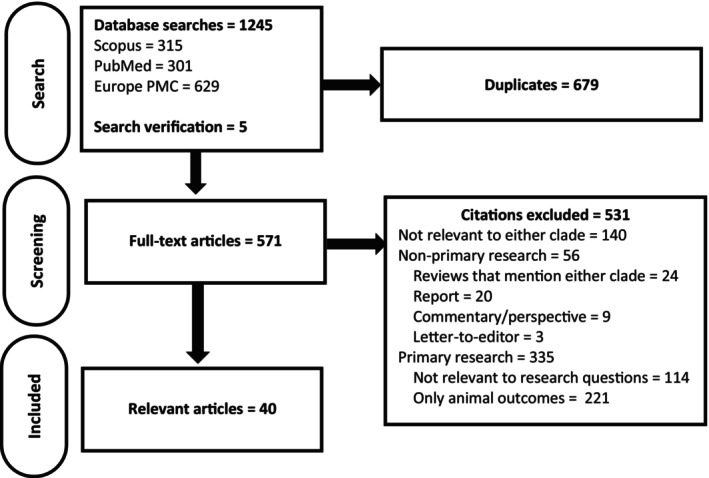
PRISMA flow diagram of articles through the rapid review process.

There were 571 full‐text articles screened for relevance, of which 40 articles were considered relevant primary research on avian influenza clades 2.3.4.4b and/or 2.3.2.1c in humans (Figure 1).

Articles were published between 2014 and 2023 with 80.0% (32/40) published since 2021, Table 1. The majority of articles were considered published primary peer‐reviewed research articles (45%; 18/40) and were conducted in Asia (57.5%; 23/40). Some articles included more than one study design, which included observational (47.5%; 19/40) and descriptive (60.0%; 24/40) study designs.

**TABLE 1:** General characteristics of 40 primary research publications on avian influenza A(H5Nx) clades 2.3.4.4b or 2.3.2.1c in humans.

Category	Count
Type of document	
Primary peer‐reviewed research	18
Short communications	11
Letter to the editor with data	3
Preprint	2
Commentary with data	1
Government Report	5
Continent	
Asia	23
Europe	7
North America	5
South America	3
Africa	2
Date of publication	
2014–2020	8
2021–2023	32
Study design^a^	
Observational	19
Case report/series	14
Surveillance or monitoring programme	1
Prospective cohort	2
Longitudinal	1
Cross‐sectional	1
Descriptive	24
Outbreak investigation	10
In vitro	8
In silico	4
Prevalence	1
Biological monitoring	1

^a^ Total number sums to > 40 as some studies used more than one study design.

## 4 Question 1: Is There a Difference in Animal‐To‐Human Transmissibility Between Avian Influenza Clade 2.3.4.4b and 2.3.2.1c Viruses?

### 4.1 Transmission to Humans/Human Exposure

Twenty‐six studies reported findings relevant to transmission or exposure of avian influenza clades 2.3.4.4b or 2.3.2.1c to humans (Table 2). ROB was assessed for 16 studies using standardised tools for the following study designs: nine case reports (Bruno et al. 2023; Castillo et al. 2023; Li et al. 2022; Oliver et al. 2022; Takayama et al. 2016; World Health Organization 2023a; World Health Organization 2023b; World Health Organization 2023c; Zhang et al. 2022), two case series (World Health Organization 2023e; Xiao et al. 2021), one prevalence study (Gomaa et al. 2023), one longitudinal study (Horwood et al. 2023), one cross‐sectional study (Ilyicheva et al. 2021) and two prospective cohort studies (Ma et al. 2018; Quan et al. 2019) (Data S2). Case reports and case series are uncontrolled studies that are all at a high ROB due to their sample methodology. They were evaluated for complete and appropriate reporting for which case reports met a median of 5/8 reporting items (range 2–6) and the two case series met 4/10 (World Health Organization 2023e) and 7/10 (Xiao et al. 2021) reporting items. The prevalence study and longitudinal study were evaluated for the appropriateness of their sampling frame and complete reporting for which they met only 3/9 (Gomaa et al. 2023) and 6/9 (Horwood et al. 2023) checklist items, respectively. The cross‐sectional study (Ilyicheva et al. 2021) was assessed to be at high ROB and the two cohort studies as medium (Quan et al. 2019) and low (Ma et al. 2018) ROB.

### 4.2 Human‐To‐Human Transmission

A lack of transmission from clade 2.3.4.4b infected cases (*n* = 12) to close contacts was reported across nine studies (Aznar et al. 2023; Bruno et al. 2023; Castillo et al. 2023; Li et al. 2022; Oliver et al. 2022; Pardo‐Roa et al. 2023; Su et al. 2022; UK Health Security Agency 2023; World Health Organization 2023c). There was one pair of cases for clade 2.3.2.1c in which no transmission occurred from an infected paediatric case to close contacts; however, the father of the paediatric case also tested positive (World Health Organization 2023b). The investigation concluded the source of exposure was likely infected poultry for both cases.

### 4.3 Animal‐To‐Human Transmission

Fifteen studies investigated the source of AIVs for human A(H5Nx) clade 2.3.4.4b infections. Four studies confirmed clade 2.3.4.4b in humans as well as in live and/or dead poultry that the cases had close contact with, providing strong evidence for animal‐to‐human transmission (Jiang et al. 2022; Li et al. 2022; Oliver et al. 2022; Pyankova et al. 2021). One study reported two PCR positive cases among workers (*n* = 133) on an infected poultry farm, but due to a lack of symptoms, low viral load and no specific H5 antibodies against the A(H5) virus, the authors concluded the positive results were most likely due to environmental contamination of the samples (Aznar et al. 2023). No transmission occurred from confirmed 2.3.4.4b infected poultry to exposed poultry farm owners/workers (*n* = 36) in two additional studies (Kniss et al. 2023; Moreno et al. 2023). In seven studies of confirmed human cases of A(H5Nx) 2.3.4.4b, one case was exposed to a bird contaminated environment (Castillo et al. 2023; Pardo‐Roa et al. 2023), five cases had direct contact with A(H5N1) infected poultry for which the clade was not confirmed/reported (UK Health Security Agency 2023; World Health Organization 2023c) and three cases had direct contact with diseased poultry or waterfowl that were not tested for AIV (Bruno et al. 2023; Xiao et al. 2021; Zhang et al. 2022). The epidemiological investigations suggested the infected/sick birds were the likely source of AIV.

Four studies investigated the source of transmission for human clade 2.3.2.1c infections. In one study, no individuals (0/663) exposed to dead or dying poultry in a farm or market were positive for AIV by RT‐qPCR (Monamele et al. 2019). Five confirmed A(H5N1) clade 2.3.2.1c cases had direct contact with diseased poultry, which were not tested for AIV (Takayama et al. 2016; World Health Organization 2023a, 2023e). The epidemiological investigations suggested the sick birds were the most likely source of transmission.

Due to the limited evidence on transmission of A(H5Nx) clade 2.3.4.4b or 2.3.2.1c to humans, it is not possible to discern differences in the likely mode of exposure, transmission or the potential of AIV infection in humans between the two clades.

### 4.4 Seropositivity and Seroconversion

Three studies investigated seropositivity/seroconversion for A(H5Nx) clade 2.3.4.4b. In one study, a lack of seropositivity or seroconversion was reported among 17 persons (0% seroprevalence) in the United States exposed to confirmed A(H5N1) clade 2.3.4.4b infected poultry (Kobasa et al. 2023). A longitudinal study of live poultry market workers in Egypt found 4.6% (38/830) of samples had neutralising antibodies against 2.3.4.4b and 8% (13/164) of the individuals who provided more than one sample seroconverted over the course of the study (between February 2022 and March 2023) (Gomaa et al. 2023). Another study reported positive serology for A(H5N2) clade 2.3.4.4b (hemagglutination inhibition (HI) titre ≥ 40) among three different groups in Vietnam to be 2% (1/52) among individuals from private households in a province where poultry deaths had been reported, 4% (4/96) among healthy blood donors, and 41% (60/147) among hospital patients with non‐communicable diseases (Ilyicheva et al. 2021). Only one of these individuals was positive by microneutralisation assay (MN) and the authors suggest that the high HI positivity in the control groups may be due to cross reactivity. One of the hospitalised patients also had a positive result for neutralising antibodies against 2.3.4.4b. It is not clear why so many of the serum samples for the latter group were positive by HI (but not MN).

Five studies investigated seropositivity/seroconversion for A(H5Nx) clade 2.3.2.1c. In a Russian study, 3.7% (28/760) of sera from people who had contact with infected or dead birds demonstrated the presence of antibodies to A(H5N1) clade 2.3.2.1c but did not report having symptoms (Ilyicheva et al. 2018). In a Chinese study, seroprevalence was 1.3% (*n* = 27) among 2124 poultry workers, which was not significantly different to 1.4% (3/216) in the control group of outpatients with non‐infectious disease at a hospital (Quan et al. 2019). Among 652 poultry workers with paired/serial serum samples, one (0.2%) seroconverted and six (0.9%) were persistently positive over four time points between December 2014 and April 2016 (Quan et al. 2019). Among individuals that were exposed to dead or dying poultry in a farm or market in Cameroon, antibodies were identified at two‐week follow‐up for 12.2% (16/131) HI titre ≥ 10, 2.3% (3/131) with HI titre ≥ 20 and 1.5% (2/131) with a four‐fold increase suggesting seroconversion (Monamele et al. 2019). One study found poultry workers in China had a seroprevalence of 1.87% (18/964), and seroconversion was 1.5% (7/468), which was significantly higher than that of swine workers (0%) and controls (0%) (Ma et al. 2018). Risk factors for A(H5N1) clade 2.3.4.4b virus seropositivity or seroconversion among poultry workers were female sex (adjusted OR: 5.48, 95% CI 2.38–12.63) and exposure to pigeons (ref. chickens) (adjusted OR: 3.13, 95% CI 1.23–8.00) (Ma et al. 2018).

There is no discernible difference in seropositivity or seroconversion between the two clades apart from one outlier study (Ilyicheva et al. 2021). Seropositivity and seroconversion were demonstrated among poultry workers and people exposed to sick or dying poultry who reported no clinical symptoms in studies for both clades, suggesting that some asymptomatic infections occur.

### 4.5 Investigation Into Human Exposure

One study had vendors wear an air sampler for 30 min at live bird markets in Cambodia (Horwood et al. 2023). They found that detection of AIV was high (10/10 samples RT‐PCR positive and 5/10 samples were positive for live virus) during periods of high AIV circulation in the bird population (February); five of these samples were confirmed to be A(H5Nx) clade 2.3.2.1c, and no samples were positive during the low period of AIV circulation (May) (Horwood et al. 2023). These findings suggest high levels of exposure to A(H5Nx) clade 2.3.2.1c for humans that spend > 30 min in a bird market during periods of high AIV circulation in birds.

**TABLE 2:** Transmission and exposure data for avian influenza A(H5Nx) clades 2.3.4.4b (*n* = 17 studies) and 2.3.2.1c (*n* = 9 studies) in humans.

Reference Study design Country Study date	Population Genotype Isolate name (if provided)	Confirmation	Outcome	Quality assessment tool and assessment result
2.3.4.4b				
Gomaa et al. (2023) Prevalence study Egypt 02–2022 to 03–2023	Live poultry market workers (*n* = 830)	NR	Seropositivity/seroconversion: 38/830 (4.6%) of the samples had neutralising antibodies with titres ranging from 1:40 to 1:320. Seropositivity was associated with gender as 5.5% of the male sera had antibodies compared with 0.6% of the females (χ2 *p* = 0.007). 13/164 (7.9%) of the individuals who provided more than one sample seroconverted from negative to positive over the course of the study. Human seropositivity appeared to follow virus detection in wild and domestic birds sold live in the markets suggesting this as a source of virus exposure	JBI prevalence “Yes” in 3/9 domains
Ilyicheva et al. (2021) Cross‐sectional Vietnam 2017 to 2018	Blood samples collected from three groups in Vietnam: private households in Nam Dinh province, where poultry deaths have been reported (2017) (*n* = 52) healthy donors (2017) (*n* = 96) patients with non‐communicable diseases in hospitals in Hanoi (2018) (*n* = 147)	HI and MN assay against A/chicken/Kostroma/1718/2017 (A(H5N2)) from clade 2.3.4.4b	Seropositivity/seroconversion: 65/295 (22%) showed a positive serum result via HI test private households where poultry deaths have been reported (1.9%, *n* = 1/52) healthy donors (4.2%, *n* = 4/96) the group with the highest level of HI positive titres (≥ 40) were among hospital patients with non‐communicable diseases (40.8% HI positive, *n* = 60/147) 1/295 (0.33%) had a positive result for neutralising antibodies against A/chicken/Kostroma/1718/2017 (A(H5N2)) from 2.3.4.4b. This person was sampled from hospitalised patients with non‐communicable diseases	NOS Cross‐sectional HIGH ROB
Castillo et al. (2023) & Pardo‐Roa et al. (2023) Outbreak investigation and case report Chile 12–2022 to 03–2023	53‐year‐old male (A(H5N1), A/Chile/25945/2023), three family contacts and nine healthcare contacts	Case: RT‐PCR (Influenza A subtype H5 lineage, specific 2.3.4.4 clade) and NGS Contacts: RT‐PCR (Influenza A subtype H5 lineage, specific 2.3.4.4 clade)	Epi investigation into *source of transmission* to case: The most probable mode of transmission for this confirmed human case was through environmental exposure, considering the significant presence of deceased sea mammals and wild birds near the patient's residence, which is within 150 m of the beach *Lack of human‐to‐human transmission* from case to contacts: Close contacts of the patient were identified, did not present clinical signs and tested negative for influenza. They completed the monitoring period successfully. All healthcare workers tested negative for influenza and concluded their monitoring period	NA JBI case report “Yes” in 3/8 domains
UK Health Security Agency (2023) Outbreak investigation United Kingdom 2023	144 individuals with exposure to infected birds including 83 (57.6%) staff directly involved in the culling operation, 30 (20.8%) farm staff, 11 (7.6%) veterinarians and 20 (13.9%) others such as health and safety managers and volunteers (four positive for 2.3.4.4b; A(H5N1), UK/2023/001, UK/2023/002, UK/2023/003, UK/2023/004) and household and contacts from the infected premises of UK/2023/003 and UK/2023/004 (*n* = 55)	PCR for A(H5N1) 2.3.4.4b	Epi investigation into *human exposure*: Four human detections of influenza A(H5N1) clade 2.3.4.4b have been confirmed on three premises with confirmed A(H5N1) infected birds. No viral sequences from birds on the linked infected premises are available to date, so clade could not be confirmed. Investigation suspected the exposure was from AIV infected birds to humans. One of the new detections is difficult to interpret due to lack of information on sample timing and may be consistent with infection or contamination of the respiratory tract. The second new detection is likely to represent contamination Lack of human‐to‐human transmission: All close contacts remained asymptomatic and tested negative. No human‐to‐human transmission occurred	NA
Kobasa et al. (2023) Outbreak investigation United States of America 02–2022 to 09–2022	People (*n* = 22) exposed (e.g., flock owners, farm workers and cullers) to commercial poultry, backyard flocks, wild birds and the environments of birds infected with A(H5N1) 2.3.4.4b	RT‐PCR against A(H5) Serological testing: 17 people with paired serum samples underwent HI and MN assay against 2.3.4.4.b A(H5N1) virus	Lack of animal‐to‐human transmission: 0/22 people tested positive for A(H5) by RT‐PCR. Lack of seropositivity/seroconversion: 0/17 persons demonstrated an increase in antibody titres to influenza A(H5) 2.3.4.4b virus	NA
Moreno et al. (2023) Outbreak investigation Italy 04–2023 to 06–2023	Farm owner, their five family members and four professionally exposed persons, in particular three veterinarians and one worker involved in bird culling and carcass disposal	RT‐PCR (A(H5N1)), ELISA (A(H5) antibodies) and MN assay (against A/duck/Italy/326224/2/22VIR909/2022 2.3.4.4b clade)	Lack of animal‐to‐human transmission: All 10 humans that had exposure to confirmed infected 2.3.4.4b hens, as well as cats and dogs with serological evidence of A(H5N1) infection, tested negative and were asymptomatic at two time points 6 to 9 weeks apart	NA
Aznar et al. (2023) Outbreak investigation Spain 01–2022 to 01–2023	Farm workers (*n* = 133) exposed to A(H5N1) poultry across 12 premises in Spain	RT‐PCR (influenza A(H5N1)), serology (specific A(H5) antibodies), WGS	Animal to human transmission: 2/133 (1.5%) workers tested positive via PCR and the viral genomes were highly related to those obtained from infected hens on the same farm. However, both workers were asymptomatic, had very low viral load and had no specific H5 antibodies against the A(H5) virus. The authors state that positive results in the PCR were most likely due to environmental contamination they do not believe these were true infections Lack of human‐to‐human transmission: Contact tracing identified one household contact for worker 1 and two for worker 2. All three contacts remained asymptomatic during the 10‐day follow‐up and tested negative by PCR	NA
Su et al. (2022) & Jiang et al. (2022) Outbreak investigation China 06–2021 to 07–2021	66‐year‐old male farmer (A(H5N6), A/Chongqing/02/2021) and close contacts (*n* = NR)	Case: RT‐PCR (AIV A(H5N6)), WGS, Sanger Sequencing Contacts: RT‐PCR (AIV A(H5N6))	Evidence of animal‐to‐human transmission: Eight backyard chickens successively became ill in June 2021. His wife slaughtered these sick chickens, then he cooked and ate these chickens within 10 days before disease onset. He also visited the local live poultry market within 10 days before disease onset. Contact with sick or dead poultry and visiting live poultry markets likely contributed to his infection. There were A(H5N6) or A(H5) positive environment samples on the premises where the patient lived or visited 10 days before disease onset. Positive percentage of A(H5N6) was 11.1% (2/18) and 33.3% (1/3) in his family premise and the live poultry market he visited, respectively. Avian influenza A(H5N6) clade 2.3.4.4b virus was isolated from poultry at the poultry market the confirmed case had visited. Most genes of the avian and human H5N6 isolates were closely related Lack of human‐to‐human transmission: No close contacts developed respiratory symptoms or tested positive for influenza A(H5N6) by RT‐PCR during follow‐up	NA
Pyankova et al. (2021) Outbreak investigation Russia 12–2020	Human case (A(H5N8), A/Astrakhan/3212/2020) and fellow poultry workers (*n* = 56)	RT‐PCR (influenza A(H5) viral RNA), WGS, HI assay (against HA gene with that of other clade 2.3.4.4b viruses detected in poultry and wild birds from 2016 to 2021 in Russia)	Evidence of animal‐to‐human transmission: Of 56 poultry workers with exposure to sick poultry, seven human isolates were positive for A(H5N8) and one was confirmed 2.3.4.4b. Five chickens were also sequenced and confirmed 2.3.4.4b. The absence of seroconversion observed in 2/5 (40%) cases could be explained by either insufficient sensitivity of the detection methods or may be an occurrence of nasal carriage or local virus replication Note: All family members remained asymptomatic through the follow‐up	NA
Bruno et al. (2023) Case report Ecuador 12–2022 to 01–2023	Nine‐year‐old girl (A(H5N1), A/human/Ecuador/01/2023) and her family members	RT‐qPCR Influenza A(H5) (Asian Lineage) and genomic sequencing	Epi investigation into *source of transmission* to case: A 2.3.4.4b infected paediatric girl was in contact with backyard poultry that died without apparent cause. Moreover, several incidents of dead backyard poultry have been reported in Bolivar Province. These were close to the origin of A(H5N1) clade 2.3.4.4b outbreak in Ecuador Lack of human‐to‐human transmission: No family members tested positive for A(H5) influenza	JBI case report “Yes” in 2/8 domains
World Health Organization (2023c) Case report United States of America 04–2022	A confirmed A(H5N1) case that participated in slaughtering poultry at a commercial poultry facility in Colorado where influenza A (H5N1) virus had been confirmed in the poultry. Nine samples were also collected from close contacts of the case and persons who participated in culling of poultry at the same facility	RT‐PCR for Influenza A(H5) and sequence analysis for subtype N1 Note: Clade is reported in CDC AIV timeline: A(H5N1) 2.3.4.4b in birds preceded this human A(H5N1) case. Implication is that this case is 2.3.4.4b	Epi investigation into *human exposure*: Confirmed case became ill while culling poultry at a farm where influenza A(H5N1) virus was confirmed in the poultry. Suspected exposure was from the sick poultry, but they were not tested for clade Lack of human‐to‐human transmission: All contact samples tested negative for influenza at two timepoints 1‐week apart	JBI case report “Yes” in 5/8 domains
Zhang et al. (2022) Case report China 01–2022 to 02–2022	Six‐year‐old girl (A(H5N6), A/Yangzhou/125/2022)	PCR for influenza A viruses, NGS, virus isolation	Epi investigation into *source of transmission* to case: There is a large inner lake connected to the Baita River in front of the paediatric case's house. Many wild waterfowl and domestic ducks are living together in the lake. Before the illness onset, she often liked to go to the lake for watching wild waterfowl and domestic ducks. Even in her house, wild bird droppings were everywhere. Moreover, during the illness onset, her pet dog died, but no epidemiological diagnosis for the pet dog was done. Her family had no poultry and did not handle poultry from markets. Four months after the occurrence of the case, one isolate of A(H5N6) virus highly homologous to the case virus was isolated from the faeces of local wild waterfowls where the case occurred, suggesting that wild waterfowls may be the direct source of this infection Note: No signs of influenza‐like symptoms in all close contacts were observed during her illness	JBI case report “Yes” in 6/8 domains
Li et al. (2022) Case report China 12–2021 to 01–2022	51‐year‐old female (A(H5N6), HZ/01) and close contacts of the patient, including seven family members, colleagues and poultry market workers (*n* = 24)	PCR for A(H5N6) influenza A, Sanger sequencing	Evidence of animal‐to‐human transmission: Of 29 samples from the poultry market the case visited, five (17.2%) were positive for A(H5N6). 7/20 (35%) samples collected from the home of the patient were positive for the A(H5N6) virus including a sample collected from the peritoneal lavage fluid of a slaughtered chicken which had the highest viral load of the environmental samples. Seven complete viral genomes (one from the patient (HZ/01) and six from the environment linked to the case) were confirmed as clade 2.3.4.4b. Isolates collected from the patient's living space were closely related to that collected from the patient, suggesting that the freshly slaughtered poultry may have been the direct source of infection, not the contaminated stalls of the poultry market Lack of human‐to‐human transmission: None of the close contacts developed symptoms, and all tested negative	JBI case report “Yes” in 5/8 domains
Oliver et al. (2022) Case report United Kingdom 12–2021	80‐year‐old male duck flock owner (A(H5N1), A/England/215201407/2021) and 11 close contacts	RT‐PCR for influenza A and WGS	Evidence of animal‐to‐human transmission: Close contact with avian influenza A(H5N1)‐infected birds (one bird confirmed 2.3.4.4b), heavily contaminated environment and lack of plausible human contacts for seasonal influenza Lack of human‐to‐human transmission: Of the 11 contacts identified (one without PPE and 10 with PPE use around the contact) were all asymptomatic at the time of swabbing and completed active monitoring for 10 days without developing symptoms. Four individuals submitted swabs and tested negative	JBI case report “Yes” in 6/8 domains
Xiao et al. (2021) Case series China 05–2021 to 07–2021	51‐year‐old female (A(H5N6), A/Sichuan/06681/2021) and 55‐year‐old man (A(H5N6), A/SiChuan‐Bazhong/1/2021)	qPCR and sequencing	Epi investigation into *source of transmission* to cases: Patients had been exposed to live poultry before their disease onset. Both lived in rural areas and raised chickens, ducks and geese in their backyard for self‐consumption. Case 1 visited the live poultry markets and purchased poultry before symptom onset. Case 2 had no history of visiting live poultry market. There were dead poultry that were farmed by both cases, and they had contact with or ate these dead poultry before their symptom onset. The poultry feeding settings of both cases were tested, with positive results in the environment samples related to both (clade not reported)	JBI case series “Yes” in 7/10 domains
2.3.2.1c				
Quan et al. (2019) Prospective cohort China 12–2014 to 04–2016	Poultry workers (*n* = 2124) who repeatedly are exposed to poultry and work in wholesale or retail live poultry markets or in backyard farms, including wholesale sellers, retail sellers, transporters, processors or feeders from seven sites in China Plus a control group (*n* = 216) of outpatients with non‐infectious diseases on physical examination at a general hospital	HI and MN assay against 2.3.2.1c A/chicken/Shanghai/02.12 HZ199‐P/2015 (SH199) Whole blood samples collected at an initial visit in December 2014 and again during three consecutive follow‐up visits in April 2015, December 2015 and April 2016	Seropositivity/seroconversion: Detected A(H5) subtypes included A(H5N1)‐SH199 clade 2.3.2.1c in 5.3% of samples from Shandong province Seroprevalence for A(H5N1)‐SH199 was 1.3% (95% CI 0.8–1.8) in poultry workers and 1.4% (3/216) in control group. No statistically significant differences in the prevalence of antibodies against other AIV subtypes between the control group and poultry workers (*p* = 0.2) Among 652 poultry workers with paired or serial serum samples during the study, one (0.2%, 95% CI 0–0.9) seroconverted and six (0.9%, 95% CI 0.3–2.0) were persistently positive	NOS cohort MEDIUM ROB (fair quality)
Ma et al. (2018) Prospective cohort China 07–2013 to 09–2016	Poultry workers, swine workers and general population controls In July 2013: Poultry workers (*n* = 511) and swine workers (*n* = 569) Controls (*n* = 915) Of these original 1995 participants, 1137 were followed up at year one (July 2014), 892 at year two (July 2015) and 701 at year three (July 2016). To compensate for the number of participants lost to follow‐up, they enrolled an additional 866 participants in July 2014, 603 in July 2015 and 124 in July 2016. New participants enrolled in 2014 were also followed in 2015 (*n* = 396) and 2016 (*n* = 339), and new participants enrolled in 2015 were followed in 2016 (*n* = 479)	HI and MN assay against clade 2.3.2.1c A(H5N1) virus (A/chicken/Jiangsu/WX927/2013) Analysis adjusted by sex and age group or variables with *p* values < 0.05	Seroprevalence/seroconversion: The overall seroprevalence of A(H5N1) viruses in poultry workers was significantly higher than in swine workers and controls (*p* < 0.05). One was positive in 2013, none were positive in 2014 or 2015 and 17 were positive in 2016. This represents a significant increase in seroprevalence of 3.46% for A(H5N1) virus among poultry workers in the 2016 survey, compared with the previous year's survey Seroprevalence Poultry workers: 18/964 (1.87% [95% CI 1.11–2.9]), *p* < 0.001 Swine workers: 0/1079 (0% [95% CI 0–0.34]), *p* < 0.001 Controls: 0/1545 (0% [95% CI0–0.24]), *p* < 0.001 Seroconversion Poultry workers: 7/468 (1.5% [95% CI 0.60–3.06]) Swine workers: 0/514 (0% [95% CI 0–0.72]) Controls: 0/1030 (0% [95% CI 0–0.36]) Sero incidence Poultry workers: 4.46/1000 person‐years (95% CI 1.80–9.17) Swine workers: 0 (95% CI 0–2.36) Controls: 0 (95% CI 0–1.43) Among poultry workers, female sex (reference male) and exposure to pigeons (reference chickens) were significant risk factors for A(H5N1) virus seropositivity or seroconversion Measures of Association Sex: Crude OR 4.07 (95% CI 1.32–12.56), Adjusted OR 5.48 (95% CI 2.38–12.63) Exposure (goose; ref. chicken): Crude OR 3.24 (95% CI 1.11–9.42), Adjusted OR 2.64 (95% CI 0.72–9.74) Exposure (pigeon; ref. chicken): Crude OR 2.85 (95% CI 1.06–7.70), Adjusted OR 3.13 (95% CI 1.23–8.00) Exposure (duck; ref. chicken): Crude OR 2.06 (95% CI 0.81–5.23), Adjusted OR 1.87 (95% CI 0.77–5.01)	NOS cohort LOW ROB (good quality)
Ilyicheva et al. (2018) Outbreak investigation Russia 11–2016 to 03–2017	People (*n* = 760) who had contact with infected or perished birds	HI and MN assay against A/rook/Chany/32/2015 (A(H5N1)) 2.3.2.1c	Seropositivity/seroconversion: There was no clinical evidence of human disease; however, 28/760 (3.7%) sera from people who had contact with infected or perished birds demonstrated the presence of antibodies to A(H5N1) virus of clade 2.3.2.1c	NA
Monamele et al. (2019) Outbreak investigation Cameroon 05–2016 to 03–2017	People (*n* = 663) were sampled for exposure to avian influenza based on presence on a farm or in a market where dead or dying poultry was identified (active infection testing)	RT‐qPCR (tested for influenza A and B) Serological testing: 131 people were sampled twice (once at baseline and 2 weeks later). Serum samples were tested for A(H5N1) clade 2.3.2.1c antibodies using HI assay and confirmed with MN assay	Lack of animal‐to‐human transmission: 0/663 people tested positive by RT‐qPCR Seropositivity/seroconversion: For the 131 tested for serological analysis, 16 (12.2%) had the possible presence of antibodies against A(H5N1) isolates from Cameroon with HI titre ≥ 10 at the 2‐week follow‐up test, three people had HI titre ≥ 20 at the 2‐week follow‐up test and two out 131 (1.5%) had a four‐fold increase between baseline and a collection 2 weeks later indicating seroconversion	NA
World Health Organization (2023a) Case report Cambodia 11–2023	Two female cases, one in the 20–25 years age group and the other less than 5 years old from the same village in Kampot Province (A(H5N1))	RT‐PCR for influenza A(H5N1) and genomic sequencing	Epi investigation into *source of transmission* to cases: Both cases had exposure to backyard birds which were reported to be sick and dead, over the past month. They both resided in the same village. The sequences cluster most closely with the viruses from the two human cases reported in October 2023. In these two cases, while human‐to‐human transmission cannot be ruled out, it is likely there were separate exposures to the viruses from sick and dead chickens	JBI case report “Yes” in 4/8 domains
World Health Organization (2023e) Case Series Cambodia 10–2023 to 11–2023	A 50‐year‐old male in Svay Rieng province and a two‐year‐old girl in Prey Veng province (A(H5N1))	Not reported	Epi investigation into *source of transmission* to cases: 50‐year‐old‐male had exposure to sick and dead chickens before his illness onset. There were reports of sick and dead poultry in the village where the two‐year‐old girl lived, and her family handled and cooked sick and dead poultry in the weeks prior to her illness onset. Information suggests separate spillover events from infected poultry to the two human cases Note: No further cases were detected among the contacts of these two cases (no testing information provided for contacts)	JBI case series “Yes” in 4/10 domains
World Health Organization (2023b) Case report Cambodia 02–2023	11‐year‐old girl (A(H5N1)) from Prey Veng province and 12 close contacts (eight asymptomatic close contacts and four symptomatic)	RT‐PCR for avian influenza A(H5N1) virus and genome sequencing	Lack of human‐to‐human transmission: One of the close contacts (her father) tested positive. All the other close contacts tested negative. An outbreak investigation is on‐going including determining the source of exposure of these two reported cases to the virus	JBI case report “Yes” in 3/8 domains
Takayama et al. (2016) Case report Vietnam 01–2014	52‐year‐old man (A(H5N1), A/Vietnam/14011801/2014)	RT‐PCR for A(H5N1) virus, WGS and virus isolation	Epi investigation into *source of transmission* to case: Exposure to dead poultry infected with A(H5N1) viruses were found scattered near his house and he buried his two dead chickens. No poultry samples were taken, so the human isolate was not verified to match that of the dead poultry	JBI case report “Yes” in 5/8 domains
Horwood et al. (2023) Longitudinal and biological monitoring study Cambodia 02–2016 to 05–2016	Environment samples only (*n* = 10) Air was tested at live bird markets (LBM)	To test the air at LBM, two LBM vendors agreed to wear a Sioutas Personal Cascade Impactor Sampler (SKC Inc.) each day of collection for 30 min while continuing with their usual activities in the LBM. Samples were collected over 5 days	Investigation into *human exposure*: Of the 10 AIV aerosol samples (five of which were identified to be A(H5N1) 2.3.2.1c) detected in filter apparatus significantly more frequently during the peak season in February (100%) compared with the low period in May (0%). No patterns were observed that suggested AIVs were detected more frequently in the larger or smaller pore size filters. Live virus was recovered by egg‐based amplification from the air filter apparatus of 50% of participants from samples collected during peak AIV circulation (February). Influenza A(H5N1) was recovered from five (50%) of the participant samples collected during this period. Live virus was not isolated from any of the samples during the period of low circulation (May) Comparison of HA and NA sequences from air and poultry samples shows direct correlation, indicating that poultry within the LBM were the source of the viruses in the air Chicken samples were positive for Influenza A(H5) in 6/20 (30%) of high season samples (February) and 0/20 low season samples (May). Phylogenetic analysis showed five of these samples were clade 2.3.2.1c. Duck samples were positive for Influenza A(H5) in 8/20 (40%) of high season samples and 1/20 (5%) low season samples. Four of these samples were clade 2.3.2.1c Of note, study did not test the LBM vendors to identify if they were infected by AIV. This study provides evidence of human exposure to 2.3.2.1c in LBMs. Exposure is high during periods of high circulation in the bird population for humans that spend > 30 min in an LBM	JBI prevalence “Yes” in 6/9 domains

Abbreviations: CI = confidence interval, ELISA = Enzyme‐linked immunosorbent assay, HA = hemagglutinin, HI = Hemagglutination inhibition, JBI = Joanna Briggs Institute, MN = Microneutralization, NA = neuraminidase, NA = Not applicable, NGS = Next Generation Sequencing, NOS = Newcastle‐Ottawa Scale, NR = not reported, PCR = polymerase chain reaction, PPE = personal protective equipment, ROB = risk of bias, RT‐PCR = Reverse transcription polymerase chain reaction, RT‐qPCR = Reverse transcription‐quantitative polymerase chain reaction, WGS = Whole genome sequencing.

## 5 Question 2: Do the Epidemiological Characteristics of Human Cases Differ Between Avian Influenza Clade 2.3.4.4b and 2.3.2.1c Viruses?

### 5.1 Human‐To‐Human Transmissibility

Transmissibility can be measured by the basic reproductive number (*R*_0_) value and secondary attack rate (SAR) of a virus. No studies on AIV A(H5Nx) clade 2.3.4.4b or 2.3.2.1c reported these outcomes as no human‐to‐human transmission of AIVs was confirmed. Thus, transmissibility within the included studies is estimated to be approximately zero with substantial uncertainty about whether human‐to‐human transmission would have been detected if it occurred.

### 5.2 Clinical Disease in Humans and Risk Factors for Severe Outcomes

Eighteen studies reported on clinical disease caused by AIV A(H5Nx) clades 2.3.4.4b or 2.3.2.1c in humans (Table 3). ROB was assessed for 11 case reports (Bi et al. 2021; Bruno et al. 2023; Castillo et al. 2023; Li et al. 2022; Oliver et al. 2022; Pabbaraju et al. 2014; Takayama et al. 2016; World Health Organization 2023a, 2023b, 2023c; Zhang et al. 2022) and three case series (Gu et al. 2022; World Health Organization 2023e; Xiao et al. 2021) (Data S2). Case reports and case series are uncontrolled studies that are all at a high risk of bias due to their sample methodology. The case reports met a median of 4/8 reporting items (range 2–6). The case series met 4/10 (Gu et al. 2022; World Health Organization 2023e) and 7/10 (Xiao et al. 2021) reporting items. Thirty‐five A(H5Nx) clade 2.3.4.4b cases had clinical data reported and included 10 cases with the A(H5N1) genotype (Aznar et al. 2023; Bruno et al. 2023; Castillo et al. 2023; Oliver et al. 2022; Pardo‐Roa et al. 2023; UK Health Security Agency 2023; World Health Organization 2023c), 24 with A(H5N6) (Bi et al. 2021; Gu et al. 2022; Li et al. 2022; Xiao et al. 2021; Zhang et al. 2022) and one with A(H5N8) (Pyankova et al. 2021). Age of cases ranged from 1 to 72 years old and included 14 males and 14 females (sex not reported for seven cases). These cases were reported from seven countries: England (*n* = 5) (Oliver et al. 2022; UK Health Security Agency 2023), Spain (*n* = 2) (Aznar et al. 2023), Chile (*n* = 1) (Castillo et al. 2023; Pardo‐Roa et al. 2023), Ecuador (*n* = 1) (Bruno et al. 2023), United States (*n* = 1) (World Health Organization 2023c), China (*n* = 24) (Bi et al. 2021; Gu et al. 2022; Li et al. 2022; Xiao et al. 2021; Zhang et al. 2022) and Russia (*n* = 1) (Pyankova et al. 2021). Hospitalisation was a reported outcome for most A(H5Nx) clade 2.3.4.4b cases (26/35, 74%), and intensive care unit (ICU) admission was reported in 9% (3/35) of cases. Eight cases (8/35, 23%) were asymptomatic (Aznar et al. 2023; Oliver et al. 2022; Pyankova et al. 2021; UK Health Security Agency 2023). All six of the fatal cases (17%) were A(H5N6) cases from China (Gu et al. 2022; Xiao et al. 2021). Exposure information prior to illness onset was reported for 46% (16/35) of cases and included exposure to poultry (14/16, 88%), exposure to a contaminated environment (1/16, 6%) and source of exposure unknown including no contact with poultry (1/16, 6%).

Eight AIV clade 2.3.2.1c cases had clinical data reported; all of which were the A(H5N1) genotype (Pabbaraju et al. 2014; Takayama et al. 2016; World Health Organization 2023a, 2023b, 2023e). Age of cases ranged from 2 to 52 years old and included three males and four females (sex not reported for one case). These cases were reported from three countries: Cambodia (*n* = 6) (World Health Organization 2023a, 2023b, 2023e), Canada (travel from China) (*n* = 1) (Pabbaraju et al. 2014) and Vietnam (*n* = 1) (Takayama et al. 2016). Hospitalisation was a reported outcome for most A(H5N1) 2.3.2.1c cases (6/8, 75%) and ICU admission was reported in 2/8 (25%) cases. One case (1/8, 13%) was asymptomatic (World Health Organization 2023b), and six cases were fatal (6/8, 75%) (Pabbaraju et al. 2014; Takayama et al. 2016; World Health Organization 2023a, 2023b, 2023e). Exposure information prior to illness onset was reported for 6/8 (75%) cases and included exposure to poultry (5/6, 83%) and source of exposure unknown including no contact with poultry (1/6, 17%).

In summary, clades 2.3.4.4b and 2.3.2.1c had similar hospitalisation rates (74% vs. 75%). However, asymptomatic cases were higher (23% vs. 13%), while ICU admission (9% vs. 25%) and fatality (18% vs. 75%) were lower for clade 2.3.4.4b cases compared to clade 2.3.2.1c. Caution is suggested in interpreting this data as it may be an indication that clade 2.3.2.1c cases are more severe or could be an artefact due to the small number of sporadic human cases reported in the literature. This precludes drawing definitive conclusions about disease severity in humans between these clades.

**TABLE 3:** Human clinical data for avian influenza A(H5Nx) clades 2.3.4.4b (*n* = 13 studies) and 2.3.2.1c (*n* = 5 studies).

Reference Study design Country Study date	Population Exposure Genotype Isolate name (if provided)	Confirmation	Outcome	Quality assessment tool and assessment result
Clade 2.3.4.4b				
UK Health Security Agency (2023) Outbreak investigation United Kingdom 2023	Four individuals Occupationally exposed to AIV infected birds on three premises *A(H5N1)* UK/2023/001, UK/2023/002, UK/2023/003, UK/2023/004	PCR for A(H5N1) 2.3.4.4b	All four cases were asymptomatic. One case did report sore throat and myalgia, but symptoms are not definitively associated with time of positivity	NA
Oliver et al. (2022) Case report United Kingdom 12–2021	80‐year‐old male Duck flock owner with exposure to infected birds (one confirmed 2.3.4.4b) *A(H5N1)* A/England/215201407/2021	RT‐PCR for influenza A and WGS	Owner who was confirmed positive for 2.3.4.4b was *asymptomatic*	JBI case report “Yes” in 6/8 domains
Aznar et al. (2023) Outbreak investigation Spain 01–2022 to 01–2023	Two farm workers Exposed to infected A(H5N1) poultry *A(H5N1)* A/CastillaLaMancha/3739/2022 A/CastillaLaMancha/3869/2022	RT‐PCR (influenza A(H5N1)), serology (specific A(H5) antibodies), WGS	Symptoms: Both cases were *asymptomatic* Note: both workers were asymptomatic, had very low viral load and had no specific A(H5) antibodies against the A/H5 virus. The authors state that positive results in the PCR may be due to environmental contamination	NA
Pardo‐Roa et al. (2023) & Castillo et al. (2023) Outbreak investigation & Case Report Chile 12–2022 to 03–2023	53‐year‐old male Lived in proximity to a seashore where A(H5N1) infected seabirds were identified *A(H5N1)* A/Chile/25945/2023	RT‐PCR (Influenza A subtype A(H5) lineage, specific 2.3.4.4 clade) and NGS	Symptoms: *Hospitalised* with cough, sore throat, hoarseness, difficulty breathing (dyspnoea) and pneumonia	NA JBI case report “Yes” in 3/8 domains
Bruno et al. (2023) Case Report Ecuador 12–2022 to 01–2023	Nine‐year‐old girl Contact with sick backyard poultry *A(H5N1)* A/human/Ecuador/01/2023	RT‐qPCR (Influenza A/H5 (Asian Lineage)) and genomic sequencing	Symptoms: Admitted to a *hospital* due to severe flu symptoms. A few days later she was transferred to the *ICU* of a paediatric hospital due to complications with septic shock and pneumonia	JBI case report “Yes” in 2/8 domains
World Health Organization (2023c) Case report United States of America 04–2022	Human case Participated in slaughtering poultry at a commercial poultry facility in Colorado where influenza A(H5N1) virus had been confirmed in the poultry *A(H5N1)*	RT‐PCR for Influenza A(H5) and sequence analysis for subtype N1 Note: Clade is reported in CDC AIV timeline: A(H5N1) 2.3.4.4b in birds preceded this human A(H5N1) case. Implication is that this case is 2.3.4.4b	Symptoms: The patient did not report symptoms other than fatigue, was not hospitalised and has since recovered	JBI case report “Yes” in 5/8 domains
Zhang et al. (2022) Case Report China 01–2022 to 02–2022	Six‐year‐old girl Exposure to wild waterfowl around home. Four months later a waterfowl faecal isolate was collected from the area and was positive for A(H5N6). *A(H5N6)* A/Yangzhou/125/2022 (YZ125)	PCR for influenza A viruses, NGS, virus isolation	Symptoms: *Hospitalised* with fever, headache, dizziness, vomiting, walking instability, language reduction, sleep increase and lethargy. No obvious respiratory symptoms. Five days later, she rapidly developed symptoms typical of acute encephalitis including lethargy, impassive and mask‐like face, limb weakness, vertigo, tremors, aphasia, torticollis, opisthotonus, epileptic seizures and convulsion and was transferred to *ICU*. She rapidly progressed to seizures and coma (Glasgow Coma Scale score, 7) and developed frequent apnoea and irregular breathing with low oxygen saturation	JBI case report “Yes” in 6/8 domains
Gu et al. (2022) Case series, challenge trial and in vitro study (receptor binding, replication and virulence studies also reported) China 12–2020 to 12–2021	13 males and 11 females, age 1–72 Exposure history not reported *A(H5N6)* reassortants bearing the clade 2.3.4.4b HA gene of A(H5N8) viruses Only seven sequences available: A/Hunan/09911/2021 A(H5N6) A/Hunan/10117/2021 A(H5N6) A/Hangzhou/01/2021(AH5N6) A/Hunan/09285/2021 A(H5N6) A/Guangxi_guilin/11151/2021 A(H5N6) A/Sichuan/06689/2021A(H5N6) A/Sichuan/06681/2021A(H5N6)	HI assay against A/whooper swan/Shanxi/4–1/2020 (H5N8) (a clade 2.3.4.4b virus) and WGS	23 cases were *hospitalised* (1 not reported) and their conditions were *fatal* (*n* = 6), critical (*n* = 8), severe (*n* = 7), mild (*n* = 2), not reported (*n* = 1). Symptom information not reported Four of these cases are reported below (Bi et al. 2021; Li et al. 2022; Xiao et al. 2021)	JBI case series “Yes” in 4/10 domains
Li et al. (2022) Case report China 12–2021 to 01–2022	51‐year‐old female Exposure to live poultry. Contact with poultry environments and a slaughtered chicken that tested positive for 2.3.4.4b A(H5N6). *A(H5N6)* A/Hangzhou/01/2021 A(H5N6) Note: This case is also captured in (Gu et al. 2022)	PCR for A(H5N6) influenza A, Sanger sequencing	Symptoms: Initially presented with respiratory symptoms, including a high fever (39.1°C), fatigue, chills and a cough. Symptoms did not resolve in three days, and other symptoms had developed (heavy cough, shortness of breath and yellow and sticky sputum), and she was admitted to a local *hospital* and transferred to the isolation ward of the *ICU*. Diagnosed with community‐acquired severe acute interstitial pneumonia	JBI case report “Yes” in 5/8 domains
Xiao et al. (2021) Case series China 05–2021 to 07–2021	51‐year‐old female and 55‐year‐old man Exposure to live poultry that came from environments positive for A(H5N6) *A(H5N6)* A/Sichuan/06681/2021A(H5N6) A/SiChuan‐Bazhong/1/2021 A(H5N6) Note: both cases are also captured in (Gu et al. 2022)	qPCR and sequencing	The female case *died*. Both cases were *hospitalised*	JBI case series “Yes” in 7/10 domains
Bi et al. (2021) Case report China 07–2021	61‐year‐old female No contact history with live poultry *A(H5N6)* A/GXguilin/11151/2021 A(H5N6) Note: This case is also captured in (Gu et al. 2022)	RT‐PCR for AIV A(H5N6) and NGS	Symptoms: *Hospitalised* with fever with a maximum temperature of 38.5°C	JBI case report “Yes” in 3/8 domains
Pyankova et al. (2021) Outbreak investigation Russia 12–2020	Two males and five females, age 29–60, only one of these cases were confirmed 2.3.3.4b (28‐year‐old female worker) Poultry workers with contact with infected 2.3.4.4b poultry *A(H5N8)* One isolate: A/Astrakhan/3212/2020	RT‐PCR (influenza A(H5) viral RNA), focus reduction neutralisation assay, HI assay against HA gene with that of other clade 2.3.4.4b viruses detected in poultry and wild birds from 2016 to 2021 in Russia, biolayer interferometry, WGS for one isolate	RNA of avian influenza A(H5N8) virus was detected in nasopharyngeal swabs taken from seven poultry workers during an outbreak at a large poultry farm. Only one human case was sequenced and confirmed for 2.3.4.4b. However, the five poultry that were tested on the farm also tested positive for 2.3.4.4b, thus likely that the other cases were of the same clade. All cases remained *asymptomatic* through the follow‐up	NA
Clade 2.3.2.1c				
World Health Organization (2023a) Case report Cambodia 11–2023	Two female cases, one in the 20–25 years age group and the other less than five years old from the same village in Kampot Province Both cases had exposure to backyard birds which were reported to be sick and dead, over the past month *A(H5N1)*	RT‐PCR for influenza A(H5N1) and genomic sequencing	Symptoms: The first reported case developed fever, cough and shortness of breath, was treated at home for several days and then visited a *hospital*. The case was admitted and in *intensive care* at the hospital and *died*. The second case had fever, cough and rash. The case was transported to *hospital* and was admitted to an isolation room in the respiratory ward of the hospital and undergoing treatment	JBI case report “Yes” in 4/8 domains
World Health Organization (2023e) Case Series Cambodia 10–2023 to 11–2023	A 50‐year‐old male in Svay Rieng province and A two‐year‐old girl in Prey Veng province Both cases had exposure to sick and dead poultry prior to illness onset A(H5N1)	Not reported	Symptoms: 50‐year‐old‐male had an onset of illness on 3 October 2023 and *died* on 7 October. Two‐year‐old girl developed illness on 3 October 2023, was admitted to *hospital* on 5 October and *died* on 6 October	JBI case series “Yes” in 4/10 domains
World Health Organization (2023b) Case report Cambodia 02–2023	11‐year‐old girl from Prey Veng province, in the south of Cambodia and her father No exposure information *A(H5N1)*	RT‐PCR for avian influenza A(H5N1) virus and genome sequencing.	Symptoms: The 11‐year‐old girl developed symptoms and received treatment at a local *hospital*. On 21 February 2023, the case was admitted to the National Paediatric Hospital with severe pneumonia. The patient *died* on 22 February 2023. The father, who was *asymptomatic*, was in isolation at the referral hospital	JBI case report “Yes” in 3/8 domains
Pabbaraju et al. (2014) Case report Canada with travel to China 12–2013 to 01–2014	Young adult No exposure to live poultry *A(H5N1)* A/Alberta/01/2014	RT‐PCR (target influenza A M gene, A(H5) subtype), Sanger sequence and viral isolation	Symptoms: malaise, chest pain, fever and pneumonia Symptom onset occurred on Dec 27, 2013, and the case presented to emergency department the next day where pneumonia was diagnosed, and they were discharged home. Five days after initial symptom onset they returned to *hospital* with worsening pleuritic chest pains and shortness of breath, a mild headache, exacerbated by head movement, right upper quadrant and epigastric pain, nausea and vomiting with no diarrhoea, and pneumonia. Individual reported visual changes and on‐going headache, and coupled with increasing oxygen requirements was *admitted to the ICU* for intubation and ventilation. Seven days post initial visit: sudden episode of tachycardia and severe hypertension followed by hypotension requiring inotropic support, dilated pupils, no pain response, encephalitis and intracranial hypertension with oedema, meningitis, ventriculitis, reduced cerebral blood flow. Ventilatory and inotropic support removed after brain death was determined (resulting in *death*)	JBI case report “Yes” in 3/8 domains
Takayama et al. (2016) Case report Vietnam 01–2014	52‐year‐old man Exposure to dead poultry infected with A(H5N1) viruses *A(H5N1)* A/Vietnam/14011801/2014	RT‐PCR for A(H5N1) virus, WGS, virus isolation	Symptoms: mild fever and general fatigue progressing to high fever. He was *hospitalised* with dyspnoea and *died* 2 days later	JBI case report “Yes” in 5/8 domains

Abbreviations: ELISA = Enzyme‐linked immunosorbent assay, HI = Hemagglutination inhibition, JBI = Joanna Briggs Institute, MN = Microneutralisation, NA = Not applicable, NGS = Next Generation Sequencing, RT‐PCR = Reverse transcription polymerase chain reaction, RT‐qPCR = Reverse transcription‐quantitative polymerase chain reaction, WGS = Whole genome sequencing.

## 6 Question 3: For Avian Influenza Clade 2.3.4.4b and 2.3.2.1c Viruses in Humans, Are There Differences in the Prevalence of Molecular Signatures Associated With Mammalian Adaptation, Transmissibility, Pathogenicity or Virulence?

### 6.1 Adaptation to Humans and Mammals

Genomic analyses of the AIV isolates from humans for both clades showed a variety of combinations of mutations associated with mammalian adaptation (Table 4, Data S3). There were three A(H5Nx) clade 2.3.2.1c studies (Pabbaraju et al. 2014; Suttie, Tok, et al. 2019; Takayama et al. 2016) and ten A(H5Nx) clade 2.3.4.4b studies (Bi et al. 2021; Ding et al. 2022; Gu et al. 2022; Li et al. 2022; Liu et al. 2022; Oliver et al. 2022; Pardo‐Roa et al. 2023; Xiao et al. 2021; Zhang et al. 2022; Zhu et al. 2022) that provided analysis of 1–19 human isolates per study. An additional study examined trends in clade 2.3.4.4b PB2 residue 627E/K/V mutations using genomic data from the Global Initiative on Sharing all Influenza Data (GISAID) (Briggs and Kapczynski 2023). One surveillance study (Zhu et al. 2022), two case series (Gu et al. 2022; Xiao et al. 2021) and six case reports (Bi et al. 2021; Li et al. 2022; Oliver et al. 2022; Pabbaraju et al. 2014; Takayama et al. 2016; Zhang et al. 2022) were eligible for ROB assessment and their results have been summarised in the epidemiology sections above and Data S2. The whole genome sequencing and phylogenetic analyses summarised in this section were not directly evaluated in the ROB assessments. Many of the identified mutations have previously been experimentally verified to understand the individual mutation's impact on the virus' characteristics which may predict the impact of the virus on different hosts (e.g., increased virulence) (Suttie, Deng, et al. 2019). This information is useful for conducting risk assessments on new isolates.

**TABLE 4:** Mutations associated with adaptation to mammals and/or humans within avian influenza A(H5Nx) isolates from humans infected with clades 2.3.2.1c (*n* = 5) and 2.3.4.4b (*n* = 24).

Mutation	Number of genomes		Adaptation	References	
	2.3.2.1c	2.3.4.4b		2.3.2.1c	2.3.4.4b
Polymerase basic 2 (PB2) protein mutations					
E627K or K627E	2	4	2.3.2.1c, one study found no adaptation and the other suggested increased virulence in mice. 2.3.4.4b, two studies reported enhanced virulence in mice and noted it is frequently identified when AIV transmits to mammals	Pabbaraju et al. (2014); Takayama et al. (2016)	Xiao et al. (2021); Zhu et al. (2022)
Q591K		3	Increased replication and virulence in mice and mammals		Pardo‐Roa et al. (2023)); Xiao et al. (2021)
D701N	1	5	2.3.2.1c, one study found no adaptation 2.3.4.4b, three studies reported enhanced virulence in mice and noted it is frequently identified when AIV transmits to mammals	Takayama et al. (2016)	Pardo‐Roa et al. (2023); Xiao et al. (2021); Zhu et al. (2022)
I292V		7	Increased replication and virulence in mammals		Gu et al. (2022))
A676T		1	Increased virulence in mice		Zhang et al. (2022)
G309D	1	8	Increased virulence in mice and mammals	Pabbaraju et al. (2014)	Gu et al. (2022); Xiao et al. (2021); Zhang et al. (2022)
I495V		1	Increased virulence in mice		Zhang et al. (2022)
K389R		7	Increased replication and virulence in mammals		Bi et al. (2021); Gu et al. (2022)
L89V	1	8	Increased replication and virulence in mice and mammals	Pabbaraju et al. (2014)	Gu et al. (2022)
R477G	1	1	Increased virulence in mice	Pabbaraju et al. (2014)	Zhang et al. (2022)
T271A		2	Enhanced viral replication in mammalian cells		Xiao et al. (2021)
T339K	1	1	Increased virulence in mice	Pabbaraju et al. (2014)	Zhang et al. (2022)
V598T		7	Increased replication and virulence in mammals		Bi et al. (2021); Gu et al. (2022)
Polymerase basic 1 (PB1) protein mutations					
P598L	1		Enhanced polymerase activity in mammalian cells and mice	Pabbaraju et al. (2014)	
P149S	1		Increased polymerase activity in mice	Pabbaraju et al. (2014)	
K357T	1		Increased polymerase activity in mice	Pabbaraju et al. (2014)	
D622G		1	Increased virulence in mice		Zhang et al. (2022)
PA protein					
S515T		1	Associated with enhanced virulence in mice		Zhang et al. (2022)
N409S		1	Increase virus replication in mammals		Bi et al. (2021)
Hemagglutinin (HA)					
158 N		8	Increased the affinity of H5 influenza virus for human‐type receptors, α–2,6 sialic acids		Ding et al. (2022); Gu et al. (2022)
186 N		8	Increased the affinity of H5 influenza virus for human‐type receptors, α–2,6 sialic acids		Ding et al. (2022); Gu et al. (2022)
225G		7	Increased replication and virulence in mammals		Gu et al. (2022)
D101N	1		Associated with improved viral fitness	Suttie, Tok, et al. (2019)	
D155N		1	Increase binding to human α‐2,6 sialic acid receptor		Bi et al. (2021)
D94N	1		Increase binding to human α‐2,6 sialic acid receptor	Suttie, Tok, et al. (2019)	
K189R	1		Increase binding to human α‐2,6 sialic acid receptor	Suttie, Tok, et al. (2019)	
K193R	1		Improved viral fitness	Suttie, Tok, et al. (2019)	
N158D	1		Improved viral fitness	Suttie, Tok, et al. (2019)	
Q222	1		Increased binding to human α‐2,6 sialic acid receptor	Pabbaraju et al. (2014)	
Q226L		2	Increased binding to human α‐2,6 sialic acid receptor		Zhu et al. (2022)
S133A	1	7	Increased binding to human α‐2,6 sialic acid receptor	Suttie, Tok, et al. (2019)	Bi et al. (2021); Liu et al. (2022)
S137A	1	8	Increased binding to human α2,6 sialic acid receptor	Suttie, Tok, et al. (2019)	Bi et al. (2021); Gu et al. (2022)
S155N	2		Increased binding to human α‐2,6 sialic acid receptor when occurring with T156A	Pabbaraju et al. (2014); Suttie, Tok, et al. (2019)	
S159N	1		Improved viral fitness	Suttie, Tok, et al. (2019)	
S227R		19	Increased binding to human α‐2,6 sialic acid receptor		Zhu et al. (2022)
T156A	3	1	Increase in specificity for α‐2,6 human‐type receptors, increased transmission in guinea pigs	Pabbaraju et al. (2014); Suttie, Tok, et al. (2019)	Bi et al. (2021)
T160A	1	9	Increased binding to human α‐2,6 sialic acid receptor	Suttie, Tok, et al. (2019)	Ding et al. (2022); Gu et al. (2022); Zhang et al. (2022)
T188I	1	7	Increased binding to human α‐2,6 sialic acid receptor	Suttie, Tok, et al. (2019)	Bi et al. (2021); Liu et al. (2022)
T192I		24	Increased binding to human α‐2,6 sialic acid receptor		Gu et al. (2022); Zhu et al. (2022)
M1 (matrix protein)					
I43M		10	Increased replication and virulence in mammals and mice		Bi et al. (2021); Gu et al. (2022); Zhang et al. (2022)
N30D	1	10	Increased replication and virulence in mammals and mice	Pabbaraju et al. (2014)	Bi et al. (2021); Gu et al. (2022); Zhang et al. (2022)
T215A	1	10	Increased replication and virulence in mammals and mice	Pabbaraju et al. (2014)	Bi et al. (2021); Gu et al. (2022); Zhang et al. (2022)
NS1, non‐structural protein 1					
V149A		1	Associated with enhanced virulence in mice		Zhang et al. (2022)
C138L		1	Associated with enhanced virulence in mice		Zhang et al. (2022)
D87E	1		Associated with increased virulence in mice	Pabbaraju et al. (2014)	
D92E	1	2	Associated with increased virulence in mice	Pabbaraju et al. (2014)	Xiao et al. (2021)
I101M	1		Associated with increased virulence in mice	Pabbaraju et al. (2014)	
I106M		14	Increased replication and virulence in mammals		Gu et al. (2022); Liu et al. (2022)
L103F		7	Increased replication and virulence in mammals		Liu et al. (2022)
L98F	1		Increased virulence in mice	Pabbaraju et al. (2014)	
P212S		2	Increased viral replication in mice		Xiao et al. (2021)
P42S	1	8	Increased replication and virulence in mammals	Pabbaraju et al. (2014)	Bi et al. (2021); Gu et al. (2022); Zhang et al. (2022)

Mutations in the HA were mainly associated with improved binding to human‐type receptors α–2,6 sialic acids, which are abundant on the surface of the epithelial cells lining the upper respiratory tract. Most AIVs are adapted to bind to sialic acids in α‐2,3 linkage, which is most abundant in the respiratory and intestinal tracts of birds but is also present in the lower respiratory tract of humans (Suttie, Deng, et al. 2019). Mutations to improve α–2,6 sialic acid binding suggest mammalian adaptation. As shown in Table 4, there were many mutation markers and little overlap in mutations (14/19 were only reported in one clade) between the clades. For the five A(H5Nx) clade 2.3.2.1c isolates, no mutation was more frequently reported compared to others. Whereas the 24 A(H5Nx) clade 2.3.4.4b isolates consistently reported S227R (*n* = 19) and T192I (*n* = 24) mutations, neither of which was reported in the clade 2.3.2.1c isolates. Four other mutations (S137A,158 N, T160A, 186 N) were reported in 33% or more of clade 2.3.4.4b isolates (Table 4).

The RNA polymerase complex (PB1, PB2 and PA) and the non‐structural protein 1 (NS1) mutations were associated with increased viral replication, polymerase activity and virulence in mammals and/or mice (Table 4). Many of the RNA polymerase complex mutations were seen in isolates from both clades, and the data does not suggest there are mutations that are dominant in either clade. Within the NS1 protein, there were two mutations associated with clade 2.3.4.4b isolates that were frequently reported: I106M (*n* = 14) and P42S (*n* = 8), with the latter also reported in an isolate from clade 2.3.2.1c.

### 6.2 Pathogenicity/Virulence in Humans

Five studies assessed pathogenicity and virulence of AIV isolates from birds and mammals infected with clades 2.3.2.1c (*n* = 1) (Yang et al. 2019) and 2.3.4.4b (*n* = 4) (Blaurock et al. 2021; Bui et al. 2021; Kobasa et al. 2023; Zhang et al. 2023) in human cell lines (Data S4). This type of in vitro experiment provides indirect evidence of the relative potential pathogenicity and virulence across studied isolates and their respective mutations. There is insufficient similar information to contrast between the clades.

The ability of AIV to replicate in human cells was the only outcome that was investigated across all five studies. The study on clade 2.3.2.1c included two isolates from chickens and one from the environment and demonstrated the virus could replicate well in both mammalian and human cells (Yang et al. 2019). The four studies on clade 2.3.4.4b all showed the ability of isolates from various bird species to replicate in human cells (Blaurock et al. 2021; Bui et al. 2021; Kobasa et al. 2023; Zhang et al. 2023). The mutations and pattern of reassortment of internal gene segments impacted the ability of two bird and two mammalian 2.3.4.4b isolates from Canada in 2021–2022 to replicate in human cells (Kobasa et al. 2023). In this study, three isolates that were different reassortants of the Eurasian and North American lineages from a red‐tailed hawk and two foxes replicated more rapidly in human cells compared to a fully Eurasian isolate from a turkey; however, there was variability in these results across human cell lines and isolates (Kobasa et al. 2023). The red‐tailed hawk isolate replicated to high virus titres in upper respiratory tract human cells (Kobasa et al. 2023). It was the most virulent and lethal isolate in white‐footed mice and was demonstrated to transmit between ferrets in an in vivo experiment (Kobasa et al. 2023).

The relative pathogenicity and virulence of AIV isolates in human cells was shown to be impacted by the host origin of the isolate (e.g., human > mammal > bird) and the optimal length of NS1, which varies by clade (Blaurock et al. 2021; Bui et al. 2021). When compared to human isolates (A(H5N1) 2.3.2.1b, A(H5N6) 2.3.4.4, A(H5N1) clade 0 and pH1N1), the replication of 2.3.4.4b isolates from birds was less efficient and induced fewer proinflammatory cytokines and chemokines in human cell lines (Bui et al. 2021).

A single study examined the HA properties of a human AIV clade 2.3.2.1c isolate from Cambodia in 2023 (Chang et al. 2023). Results demonstrated that while the virus has limited zoonotic potential and does not bind to human receptor α–2,6 sialic acids, it did have increased HA thermal stability and reduced pH of fusion (Chang et al. 2023). Reduced pH of fusion has been shown to increase transmissibility in ducks and in other AIVs was important for airborne transmission among chickens, which could improve the fitness of the virus circulating in birds (Chang et al. 2023).

## 7 Discussion

The evidence on AIV clades 2.3.2.1c and 2.3.4.4b in humans is summarised and contrasted in this RR. The findings support concerns regarding the zoonotic potential of AIVs from these clades given the propensity of AIVs to reassort and the occurrence of human and mammalian infections from the last couple of years (Food and Agricultural Organization of the United Nations 2023). This RR identified a limited number of studies with relevant data on these two clades, which may have impeded the identification of significant differences between the clades.

The AIV 2.3.2.1c clade has undergone two intercontinental waves, A(H5N1) 2009–2010 and 2014–2015 in birds, and it continues to circulate within birds in the Mekong area (Charostad et al. 2023). Whereas the 2.3.4.4b clade emerged and caused waves of A(H5Nx) in birds, starting in 2016–2017, as well as the current A(H5N1) wave in birds that started in late 2020 (Charostad et al. 2023; Food and Agricultural Organization of the United Nations 2023). Both clades are well adapted to wild migratory birds, particularly waterfowl (Charostad et al. 2023; Food and Agricultural Organization of the United Nations 2023). While there have been a limited number of human cases caused by each clade to date, the current A(H5Nx) clade 2.3.4.4b wave is a result of an unprecedented duration and number of outbreaks impacting a large range of both domestic and wild bird species across a broad geographic range, as well as an unusual number of cases in multiple mammalian species (Centers for Disease Control and Prevention 2023b; Charostad et al. 2023).

Several markers of mammalian adaptation and mutations associated with increased AIV viral replication, polymerase activity and virulence in mammals and/or mice were found in both clades. There was little overlap between the clades, and most did not appear at a high frequency for either clade. Mutations in the HA related to the binding of α–2,6 sialic acids compared to α–2,3 sialic acids, which are more common in humans and birds, respectively. The presence of mutations that favour α–2,6 sialic acids is considered to be markers of mammalian adaptation (Suttie, Deng, et al. 2019). The two HA mutations reported at a high frequency in isolates of the 2.3.4.4b clade included S227R, for which no experimental evaluation of the phenotype was identified, and T192I, that has been documented to increase binding to the α‐2,6 sialic acid receptor (Suttie, Deng, et al. 2019). Mutations in other domains PB1, PB2, PA and NS1 were mainly associated with virulence and virus replication. A few in vitro studies demonstrated that human isolates were more pathogenic/virulent and replicated more effectively compared to mammal and avian isolates with the same mutations in human cell lines (Blaurock et al. 2021; Bui et al. 2021; Kobasa et al. 2023). This suggests potential adaptation in human isolates compared to isolates from other species.

Evidence of human‐to‐human transmission has not been found for either AIV clade; estimates of transmissibility were not presented. Cases identified were mainly due to zoonotic transmission from infected birds, and infection appears to be rare even for individuals at high risk of exposure to AIVs. Seropositivity and seroconversion were similar for the two clades (0%–4.6%) among those exposed to infected poultry. The serological data suggest that some asymptomatic infections occur from exposure to AIV from both clades. It is also possible that some asymptomatic and mild cases detected by RT‐PCR could represent environmental contamination/contamination of the nasal mucosa instead of actual infection, as suggested for cases reported in two studies (Kniss et al. 2023; UK Health Security Agency 2023). There is a need for continued surveillance and large analytical epidemiological studies to determine the prevalence and burden of avian influenza in human populations.

Hospitalisation rates were found to be similar between the two AIV clades. However, the ICU admission and case fatality rates were higher for 2.3.2.1c infections, while asymptomatic infection rates were lower. These findings may be an indication that clade 2.3.2.1c may cause more severe infection in humans; however, this is based on a small sample size of mainly sporadic cases and is associated with substantial uncertainty that the few observations identified, mainly from studies considered to be at high risk of bias, are representative of human infections with this clade and, thus, should be interpreted with caution.

Within this RR we did not identify any relevant research that directly compared AIV A(H5Nx) clades 2.3.2.1c and 2.3.4.4b, which made it challenging to contrast the evidence for the two clades, particularly with the small number of included studies. The extent to which the limited number of cases reported represents human AIV cases that have occurred may be biased by variability in country‐level capacities for epidemiological surveillance and the use of different diagnostic and sample collection methods. In addition, the evidence underpinning the human impact of AIV A(H5Nx) clades 2.3.2.1c and 2.3.4.4b was largely descriptive or based on in vitro experiments, which are considered to be at high ROB and provide lower‐level evidence for answering the RR questions. Analyses of mutations may also be biased by the selection of isolates used within the genetic studies. Thus, there were limited observations within low‐quality study designs that underpin the evidence to answer our research questions, suggesting that overall there is low certainty in the findings. The results of this RR may change with additional research.

This RR was conducted to inform discussions around the current AIV panzootic and public health‐oriented risk assessments. To meet the timelines for this discussion, we chose to conduct an RR to speed up the review process. The search strategy was thorough, but only three databases were used. The search verification included reference lists of systematic reviews captured by the search and technical reports produced by major public health organisations globally. Despite not identifying any omitted published literature during the search verification process, it is possible that some research was not captured due to a lack of citation indexing in the databases examined or failure to publish. The only shortcut taken was for the data extraction and quality assessment process which was conducted by a single reviewer and verified by a second reviewer instead of two independent reviewers, which may have led to some errors in the dataset. Quality assessment was only conducted on studies for which there were tools available; thus, 50% of the studies did not undergo an assessment.

## 8 Conclusion

This RR represents a thorough review of existing evidence on the epidemiology, transmission, pathogenicity and adaptation of human AIV A(H5Nx) from clades 2.3.4.4b and 2.3.2.1c. Despite there being no evidence contrasting the clades, both appear to have low infection potential for exposed humans. However, for humans that do become infected, disease can range in severity from asymptomatic/mild to critical and fatal, particularly for those infected with 2.3.2.1c. Analysis of mammalian mutation patterns across the isolates did not suggest there were mutations that could be linked with obvious phenotypes or disease severity in humans.

Both the findings and conclusions presented represent what is known as of December 2023 and can reasonably be used to inform discussions about the AIV A(H5Nx) in humans. Due to the limited number of observations and small sample sizes across studies for either clade as well as the preliminary and descriptive study designs reporting relevant evidence, there is low confidence in the findings, and results of this RR may change with future research. Most importantly, the evidence suggests that there is on‐going adaptation pressure of the circulating AIVs and evidence of mammalian adaptation (Plaza et al. 2024). It is critical to continue surveillance programmes in birds, mammals and humans to facilitate early identification of important emerging AIVs, as well as conduct large epidemiological studies to determine the prevalence and burden of avian influenza in human populations and prepare mitigation strategies from a One Health perspective.

## Author Contributions

L.A.W., N.A., E.L., T.C.: conceptualisation; L.A.W., T.C.: methodology; T.C.: project administration; L.A.W., T.C., K.M.Y.: supervision; T.C., K.M.Y., K.P., A.B., M.Q.: data extraction; T.C., K.M.Y., L.A.W.: formal analysis; T.C., K.M.Y., L.A.W.: writing – original draft; T.C., K.M.Y., L.A.W., N.A., E.L., K.P., A.B., M.Q.: writing – review and editing.

## Conflicts of Interest

The authors declare no conflicts of interest.

## Supporting information


**Data S1:** Protocol.

**Data S2:** Full dataset, excluded studies, risk of bias.

**Table S1:** Detailed table of mutations associated with adaptation to mammals and/or humans within avian influenza isolates from humans infected with clades 2.3.2.1c and 2.3.4.4b.

**Table S2:** Pathogenicity and virulence of avian influenza isolates from birds and mammals infected with 2.3.2.1c and 2.3.4.4b in human cell lines.

## Data Availability

All relevant data are included in the paper or its Supporting Information.

## References

[zph70006-bib-0001] Aznar, E. , I. Casas , A. González Praetorius , et al. 2023. “Influenza A(H5N1) Detection in Two Asymptomatic Poultry Farm Workers in Spain, September to October 2022: Suspected Environmental Contamination.” Euro Surveillance: Bulletin Europeen Sur les Maladies Transmissibles = European Communicable Disease Bulletin 28, no. 8: 2300107. 10.2807/1560-7917.es.2023.28.8.2300107. 36820643 PMC9951258

[zph70006-bib-0002] Bi, F. , L. Jiang , L. Huang , et al. 2021. “Genetic Characterization of Two Human Cases Infected With the Avian Influenza A (H5N6) Viruses—Guangxi Zhuang Autonomous Region, China, 2021.” China CDC Weekly 3, no. 44: 923–928. 10.46234/ccdcw2021.199. 34745693 PMC8563334

[zph70006-bib-0003] Blaurock, C. , U. Blohm , C. Luttermann , et al. 2021. “The C‐Terminus of Non‐Structural Protein 1 (NS1) in H5N8 Clade 2.3.4.4 Avian Influenza Virus Affects Virus Fitness in Human Cells and Virulence in Mice.” Emerging Microbes & Infections 10, no. 1: 1760–1776. 10.1080/22221751.2021.1971568. 34420477 PMC8432360

[zph70006-bib-0004] Briggs, K. , and D. R. Kapczynski . 2023. “Comparative Analysis of PB2 Residue 627E/K/V in H5 Subtypes of Avian Influenza Viruses Isolated From Birds and Mammals.” Frontiers in Veterinary Science 10: 1250952. 10.3389/fvets.2023.1250952. 37720472 PMC10502342

[zph70006-bib-0005] Bruno, A. , A. Alfaro‐Núñez , D. de Mora , et al. 2023. “First Case of Human Infection With Highly Pathogenic H5 Avian Influenza A Virus in South America: A New Zoonotic Pandemic Threat for 2023?” Journal of Travel Medicine 30, no. 5: taad032. 10.1093/jtm/taad032. 36881656 PMC10481407

[zph70006-bib-0006] Bui, C. H. T. , D. I. T. Kuok , H. W. Yeung , et al. 2021. “Risk Assessment for Highly Pathogenic Avian Influenza A(H5N6/H5N8) Clade 2.3.4.4 Viruses.” Emerging Infectious Diseases 27, no. 10: 2619–2627. 10.3201/eid2710.210297. 34545790 PMC8462306

[zph70006-bib-0007] Castillo, A. , R. Fasce , B. Parra , et al. 2023. “The First Case of Human Infection With H5N1 Avian Influenza A Virus in Chile.” Journal of Travel Medicine 30, no. 5: taad083. 10.1093/jtm/taad083. 37310882 PMC10481412

[zph70006-bib-0008] Centers for Disease Control and Prevention . 2023a. “Emergence and Evolution of H5N1 Bird Flu.” https://www.cdc.gov/flu/avianflu/communication‐resources/bird‐flu‐origin‐infographic.html#print.

[zph70006-bib-0009] Centers for Disease Control and Prevention . 2023b. “Technical Report: Highly Pathogenic Avian Influenza A(H5N1) Viruses.” https://www.cdc.gov/flu/avianflu/spotlights/2022‐2023/h5n1‐technical‐report_december.htm.

[zph70006-bib-0010] Chang, P. , J. Yang , T. K. Karunarathna , M. Qureshi , J. Sadeyen , and M. Iqbal . 2023. “Characterization of the Haemagglutinin Properties of the H5N1 Avian Influenza Virus That Caused Human Infections in Cambodia.” Emerging Microbes & Infections 12, no. 2: 2244091. 10.1080/22221751.2023.2244091. 37526446 PMC10461499

[zph70006-bib-0011] Charostad, J. , M. Rezaei Zadeh Rukerd , S. Mahmoudvand , et al. 2023. “A Comprehensive Review of Highly Pathogenic Avian Influenza (HPAI) H5N1: An Imminent Threat at Doorstep.” Travel Medicine and Infectious Disease 55: 102638. 10.1016/j.tmaid.2023.102638. 37652253

[zph70006-bib-0012] Chauhan, R. P. , Z. G. Dessie , A. Noreddin , and M. El Zowalaty . 2020. “Systematic Review of Important Viral Diseases in Africa in Light of the ‘One Health’ Concept.” Pathogens 9: 301. 10.3390/pathogens9040301. 32325980 PMC7238228

[zph70006-bib-0013] Chauhan, R. P. , and M. L. Gordon . 2022. “A Systematic Review of Influenza A Virus Prevalence and Transmission Dynamics in Backyard Swine Populations Globally.” Porcine Health Management 8, no. 1: 10. 10.1186/s40813-022-00251-4. 35287744 PMC8919175

[zph70006-bib-0014] Ding, L. , J. Li , X. Li , and B. Qu . 2022. “Evolutionary and Mutational Characterization of the First H5N8 Subtype Influenza A Virus in Humans.” Pathogens 11: 666. 10.3390/pathogens11060666. 35745520 PMC9227545

[zph70006-bib-0015] European Food, S. A , European Centre for Disease Prevention, and Control , European Union Reference Laboratory for Avian Influenza , et al. 2023. “Avian Influenza Overview September–December 2023.” EFSA Journal 21, no. 12: e8539. 10.2903/j.efsa.2023.8539. 38116102 PMC10730024

[zph70006-bib-0016] Food and Agricultural Organization of the United Nations . 2023. Global Avian Influenza Viruses With Zoonotic Potential Situation Update. FAO. https://www.fao.org/animal‐health/situation‐updates/global‐aiv‐with‐zoonotic‐potential/en.

[zph70006-bib-0017] Garritty, C. , G. Gartlehner , B. Nussbaumer‐Streit , et al. 2021. “Cochrane Rapid Reviews Methods Group Offers Evidence‐Informed Guidance to Conduct Rapid Reviews.” Journal of Clinical Epidemiology 130: 13–22. 10.1016/j.jclinepi.2020.10.007. 33068715 PMC7557165

[zph70006-bib-0018] Garritty, C. , A. C. Tricco , M. Smith , et al. 2023. “Rapid Reviews Methods Series: Involving Patient and Public Partners, Healthcare Providers and Policymakers as Knowledge Users.” BMJ Evidence‐Based Medicine 29: 55–61. 10.1136/bmjebm-2022-112070. PMC10850627 37076265

[zph70006-bib-0019] Gartlehner, G. , B. Nussbaumer‐Streit , D. Devane , et al. 2023. “Rapid Reviews Methods Series: Guidance on Assessing the Certainty of Evidence.” BMJ Evidence‐Based Medicine 29: 50–54. 10.1136/bmjebm-2022-112111. PMC10850678 37076264

[zph70006-bib-0020] Gomaa, M. , Y. Moatasim , A. El Taweel , et al. 2023. “We Are Underestimating, Again, the True Burden of H5N1 in Humans.” BMJ Global Health 8, no. 8: e013146. 10.1136/bmjgh-2023-013146. PMC10465887 37643809

[zph70006-bib-0021] Gu, W. , J. Shi , P. Cui , et al. 2022. “Novel H5N6 Reassortants Bearing the Clade 2.3.4.4b HA Gene of H5N8 Virus Have Been Detected in Poultry and Caused Multiple Human Infections in China.” Emerging Microbes & Infections 11, no. 1: 1174–1185. 10.1080/22221751.2022.2063076. 35380505 PMC9126593

[zph70006-bib-0022] Herzog, R. , M. J. Álvarez‐Pasquin , C. Díaz , J. L. Del Barrio , J. M. Estrada , and Á. Gil . 2013. “Are Healthcare Workers' Intentions to Vaccinate Related to Their Knowledge, Beliefs and Attitudes? A Systematic Review.” BMC Public Health 13, no. 1: 154. 10.1186/1471-2458-13-154. 23421987 PMC3602084

[zph70006-bib-0023] Horwood, P. F. , S. V. Horm , S. Yann , et al. 2023. “Aerosol Exposure of Live Bird Market Workers to Viable Influenza A/H5N1 and A/H9N2 Viruses, Cambodia.” Zoonoses and Public Health 70, no. 2: 171–175. 10.1111/zph.13009. 36409285 PMC10098856

[zph70006-bib-0024] Ilyicheva, T. , V. Marchenko , O. Pyankova , et al. 2021. “Antibodies to Highly Pathogenic A/H5Nx (Clade 2.3.4.4) Influenza Viruses in the Sera of Vietnamese Residents.” Pathogens 10, no. 4: 394. 10.3390/pathogens10040394. 33806156 PMC8064466

[zph70006-bib-0025] Ilyicheva, T. N. , A. G. Durymanov , S. V. Svyatchenko , et al. 2018. “Humoral Immunity to Influenza in an At‐Risk Population and Severe Influenza Cases in Russia in 2016–2017.” Archives of Virology 163, no. 10: 2675–2685. 10.1007/s00705-018-3904-9. 29872951

[zph70006-bib-0026] Jiang, W. , C. Dong , S. Liu , et al. 2022. “Emerging Novel Reassortant Influenza A(H5N6) Viruses in Poultry and Humans, China, 2021.” Emerging Infectious Diseases 28, no. 5: 1064–1066. 10.3201/eid2805.212163. 35447059 PMC9045449

[zph70006-bib-0027] Kalonda, A. , M. Phonera , N. Saasa , et al. 2021. “Influenza A and D Viruses in Non‐Human Mammalian Hosts in Africa: A Systematic Review and Meta‐Analysis.” Viruses 13, no. 12: 2411. 10.3390/v13122411. 34960680 PMC8706448

[zph70006-bib-0028] Klerings, I. , S. Robalino , A. Booth , et al. 2023. “Rapid Reviews Methods Series: Guidance on Literature Search.” BMJ Evidence‐Based Medicine 28, no. 6: 412–417. 10.1136/bmjebm-2022-112079. PMC10715472 37076268

[zph70006-bib-0029] Kniss, K. , K. M. Sumner , K. J. Tastad , et al. 2023. “Risk for Infection in Humans After Exposure to Birds Infected With Highly Pathogenic Avian Influenza A(H5N1) Virus, United States, 2022.” Emerging Infectious Diseases 29, no. 6: 1215–1219. 10.3201/eid2906.230103. 37095080 PMC10202859

[zph70006-bib-0030] Kobasa, D. , B. Warner , T. Alkie , et al. 2023. “Transmission of Lethal H5N1 Clade 2.3.4.4b Avian Influenza in Ferrets.” *Research Square*. 10.21203/rs.3.rs-2842567/v1.

[zph70006-bib-0031] Li, J. , Y. Fang , X. Qiu , et al. 2022. “Human Infection With Avian‐Origin H5N6 Influenza a Virus After Exposure to Slaughtered Poultry.” Emerging Microbes & Infections 11, no. 1: 807–810. 10.1080/22221751.2022.2048971. 35234570 PMC8920390

[zph70006-bib-0032] Liu, H. , C. Wu , Z. Pang , R. Zhao , M. Liao , and H. Sun . 2022. “Phylogenetic and Phylogeographic Analysis of the Highly Pathogenic H5N6 Avian Influenza Virus in China.” Viruses 14, no. 8: 1752. 10.3390/v14081752. 36016374 PMC9415468

[zph70006-bib-0033] Ma, M. , T. Zhao , S. Chen , et al. 2018. “Avian Influenza A Virus Infection Among Workers at Live Poultry Markets, China, 2013–2016.” Emerging Infectious Diseases 24, no. 7: 1246–1256. 10.3201/eid2407.172059. 29912708 PMC6038753

[zph70006-bib-0034] Monamele, C. G. , P. Y , E. A. Karlsson , et al. 2019. “Evidence of Exposure and Human Seroconversion During an Outbreak of Avian Influenza A(H5N1) Among Poultry in Cameroon.” Emerging Microbes & Infections 8, no. 1: 186–196. 10.1080/22221751.2018.1564631. 30866772 PMC6455145

[zph70006-bib-0035] Moola, S. , C. Tufanaru , E. Aromataris , et al. 2020. “JBI Critical Appraisal Checklist for Case Reports.” https://jbi.global/sites/default/files/2019‐05/JBI_Critical_Appraisal‐Checklist_for_Case_Reports2017_0.pdf.

[zph70006-bib-0036] Moreno, A. , F. Bonfante , A. Bortolami , et al. 2023. “Asymptomatic Infection With Clade 2.3.4.4b Highly Pathogenic Avian Influenza A(H5N1) in Carnivore Pets, Italy, April 2023.” Euro Surveillance: Bulletin Europeen Sur les Maladies Transmissibles = European Communicable Disease Bulletin 28, no. 35: 2300441. 10.2807/1560–7917.ES.2023.28.35.2300441. 37650905 PMC10472752

[zph70006-bib-0037] Munn, Z. , T. H. Barker , S. Moola , et al. 2020. “Methodological Quality of Case Series Studies: An Introduction to the JBI Critical Appraisal Tool.” JBI Evidence Synthesis 18, no. 10: 2127–2133. 10.11124/JBISRIR-D-19-00099. 33038125

[zph70006-bib-0038] Munn, Z. , S. Moola , K. Lisy , D. Riitano , and C. Tufanaru . 2015. “Methodological Guidance for Systematic Reviews of Observational Epidemiological Studies Reporting Prevalence and Cumulative Incidence Data.” International Journal of Evidence‐Based Healthcare 13, no. 3: 147–153. 10.1097/XEB.0000000000000054. 26317388

[zph70006-bib-0039] Nussbaumer‐Streit, B. , I. Sommer , C. Hamel , et al. 2023. “Rapid Reviews Methods Series: Guidance on Team Considerations, Study Selection, Data Extraction and Risk of Bias Assessment.” BMJ Evidence‐Based Medicine 28, no. 6: 418–423. 10.1136/bmjebm-2022-112185. PMC10715469 37076266

[zph70006-bib-0040] Oliver, I. , J. Roberts , C. S. Brown , et al. 2022. “A Case of Avian Influenza A(H5N1) in England, January 2022.” Euro Surveillance: Bulletin Europeen Sur les Maladies Transmissibles = European Communicable Disease Bulletin 27, no. 5: 2200061. 10.2807/1560-7917.es.2022.27.5.2200061. 35115075 PMC8815099

[zph70006-bib-0041] Pabbaraju, K. , R. Tellier , S. Wong , et al. 2014. “Full‐Genome Analysis of Avian Influenza A(H5N1) Virus From a Human, North America, 2013.” Emerging Infectious Diseases 20, no. 5: 887–891. 10.3201/eid2005.140164. 24755439 PMC4012823

[zph70006-bib-0042] Page, M. J. , J. E. McKenzie , P. M. Bossuyt , et al. 2021. “The PRISMA 2020 Statement: An Updated Guideline for Reporting Systematic Reviews.” PLoS Medicine 18, no. 3: e1003583. 10.1371/journal.pmed.1003583. 33780438 PMC8007028

[zph70006-bib-0043] Pan American Health Organization , and World Health Organization . 2023. Epidemiological Update: Outbreaks of Avian Influenza Caused by Influenza A(H5N1) in the Region of the Americas. PAHO/WHO. https://www.paho.org/en/documents/epidemiological‐update‐outbreaks‐avian‐influenza‐caused‐influenza‐ah5n1‐region‐americas‐0.

[zph70006-bib-0044] Pardo‐Roa, C. , M. I. Nelson , N. Ariyama , et al. 2023. “Cross‐Species Transmission and PB2 Mammalian Adaptations of Highly Pathogenic Avian Influenza A/H5N1 Viruses in Chile.” *bioRxiv* The Preprint Server for Biology. 10.1101/2023.06.30.547205.

[zph70006-bib-0045] Plaza, P. I. , V. Gamarra‐Toledo , J. R. Eugui , and S. A. Lambertucci . 2024. “Recent Changes in Patterns of Mammal Infection With Highly Pathogenic Avian Influenza A(H5N1) Virus Worldwide.” Emerging Infectious Diseases 30, no. 3: 444–452. 38407173 10.3201/eid3003.231098 PMC10902543

[zph70006-bib-0046] Public Health Agency of Canada . 2023. “Human Emerging Respiratory Pathogens Bulletin: Issue 84, December 2023.” https://www.canada.ca/en/public‐health/services/surveillance/human‐emerging‐respiratory‐pathogens‐bulletin/2023/december.html.

[zph70006-bib-0047] Pyankova, O. G. , I. M. Susloparov , A. A. Moiseeva , et al. 2021. “Isolation of Clade 2.3.4.4b A(H5N8), a Highly Pathogenic Avian Influenza Virus, From a Worker During an Outbreak on a Poultry Farm, Russia, December 2020.” Euro Surveillance: Bulletin Europeen sur les Maladies Transmissibles = European Communicable Disease Bulletin 26, no. 24: 2100439. 10.2807/1560-7917.ES.2021.26.24.2100439. 34142650 PMC8212591

[zph70006-bib-0048] Quan, C. , Q. Wang , J. Zhang , et al. 2019. “Avian Influenza A Viruses Among Occupationally Exposed Populations, China, 2014–2016.” Emerging Infectious Diseases 25, no. 12: 2215–2225. 10.3201/eid2512.190261. 31742536 PMC6874249

[zph70006-bib-0049] Su, K. , Z. Yu , Y. Lan , et al. 2022. “Three Cases Infected With Avian Influenza A(H5N6) Virus—Chongqing Municipality, China, January–September, 2021.” China CDC Weekly 4, no. 1: 11–16. 10.46234/ccdcw2021.278. 35586756 PMC8796724

[zph70006-bib-0050] Suttie, A. , Y. Deng , A. R. Greenhill , P. Dussart , P. F. Horwood , and E. A. Karlsson . 2019. “Inventory of Molecular Markers Affecting Biological Characteristics of Avian Influenza A Viruses.” Virus Genes 55, no. 6: 739–768. 10.1007/s11262-019-01700-z. 31428925 PMC6831541

[zph70006-bib-0051] Suttie, A. , S. Tok , S. Yann , et al. 2019. “Diversity of A(H5N1) Clade 2.3.2.1c Avian Influenza Viruses With Evidence of Reassortment in Cambodia, 2014–2016.” PLoS One 14, no. 12: e0226108. 10.1371/journal.pone.0226108. 31815962 PMC6901219

[zph70006-bib-0052] Tahmo, N. B. , F. S. Wirsiy , D. Nnamdi , et al. 2023. “An Epidemiological Synthesis of Emerging and Re‐Emerging Zoonotic Disease Threats in Cameroon, 2000–2022: A Systematic Review.” IJID Regions 7: 109. 10.1016/j.ijregi.2022.12.001. PMC10050484 37009575

[zph70006-bib-0053] Takayama, I. , N. T. Hieu , M. Shirakura , et al. 2016. “Novel Reassortant Avian Influenza A(H5N1) Virus in Human, Southern Vietnam, 2014.” Emerging Infectious Diseases 22, no. 3: 557–559. 10.3201/eid2203.151360. 26889960 PMC4766877

[zph70006-bib-0054] UK Health Security Agency . 2023. Investigation Into the Risk to Human Health of Avian Influenza (Influenza A H5N1) in England: Technical Briefing 5. UKHSA. https://www.gov.uk/government/publications/avian‐influenza‐influenza‐a‐h5n1‐technical‐briefings/investigation‐into‐the‐risk‐to‐human‐health‐of‐avian‐influenza‐influenza‐a‐h5n1‐in‐england‐technical‐briefing‐5.

[zph70006-bib-0055] Wells, G. , D. O'Connell , J. Robertson , V. Welch , and P. Tugwell . 2021. “The Newcastle‐Ottawa Scale (NOS) for Assessing the Quality of Nonrandomised Studies in Meta‐Analyses.” https://www.ohri.ca/js/clinical_epidemiology/nosgen.pdf.

[zph70006-bib-0056] World Health Organization . 2023a. Avian influenza A (H5N1)—Cambodia. WHO. https://www.who.int/emergencies/disease‐outbreak‐news/item/2023‐DON495.

[zph70006-bib-0057] World Health Organization . 2023b. Avian influenza A (H5N1)—Cambodia. WHO. https://www.who.int/emergencies/disease‐outbreak‐news/item/2023‐DON445.

[zph70006-bib-0058] World Health Organization . 2023c. Avian Influenza A (H5N1)—United States of America. WHO. https://www.who.int/emergencies/disease‐outbreak‐news/item/2022‐DON379.

[zph70006-bib-0059] World Health Organization . 2023d. “Avian Influenza Weekly Update Number 928.” https://iris.who.int/bitstream/handle/10665/375483/AI‐20240105.pdf?sequence=1&isAllowed=y.

[zph70006-bib-0060] World Health Organization . 2023e. Influenza at the Human‐Animal Interface Summary and Risk Assessment, From 4 October to 1 November 2023. WHO. https://cdn.who.int/media/docs/default‐source/influenza/human‐animal‐interface‐risk‐assessments/influenza‐at‐the‐human‐animal‐interface‐summary‐and‐assessment‐‐from‐4‐october‐to‐1‐november‐2023.pdf?sfvrsn=6c67e7df_2&download=true.

[zph70006-bib-0061] World Health Organization . 2024. “WHO Outbreak Reports.” https://www.who.int/emergencies/disease‐outbreak‐news.

[zph70006-bib-0062] Xiao, C. , J. Xu , Y. Lan , et al. 2021. “Five Independent Cases of Human Infection With Avian Influenza H5N6—Sichuan Province, China, 2021.” China CDC Weekly 3, no. 36: 751–756. 10.46234/ccdcw2021.187. 34594983 PMC8427102

[zph70006-bib-0063] Yang, J. , Z. Wang , Y. Du , et al. 2019. “Clade 2.3.2.1 H5N1 Avian Influenza Viruses Circulate at the Interface of Migratory and Domestic Birds Around Qinghai Lake in China.” Veterinary Microbiology 235: 234–242. 10.1016/j.vetmic.2019.07.009. 31383307

[zph70006-bib-0064] Zhang, C. , Z. Wang , H. Cui , et al. 2023. “Emergence of H5N8 Avian Influenza Virus in Domestic Geese in a Wild Bird Habitat, Yishui Lake, North Central China.” Virologica Sinica 38, no. 1: 157–161. 10.1016/j.virs.2022.10.002. 36265796 PMC10006182

[zph70006-bib-0065] Zhang, L. , K. Liu , Q. Su , et al. 2022. “Clinical Features of the First Critical Case of Acute Encephalitis Caused by the Avian Influenza A (H5N6) Virus.” Emerging Microbes & Infections 11, no. 1: 2437–2446. 10.1080/22221751.2022.2122584. 36093829 PMC9621215

[zph70006-bib-0066] Zhu, W. , X. Li , J. Dong , et al. 2022. “Epidemiologic, Clinical, and Genetic Characteristics of Human Infections With Influenza A(H5N6) Viruses, China.” Emerging Infectious Diseases 28, no. 7: 1332–1344. 10.3201/eid2807.212482. 35476714 PMC9239879

