# Supplementary material for: A Rapid Review Contrasting the Evidence on Avian Influenza A(H5Nx) Clades 2.3.4.4b and 2.3.2.1c in Humans
Source: Zoonoses Public Health. 2025 Aug 26;72(7):553–86. doi: 10.1111/zph.70006 (PMC12508786; doi:10.1111/zph.70006)
Supplement: Supplementary file 1 — Data S1: Protocol. [file ZPH-72-553-s002.docx]

A rapid review contrasting the evidence on avian influenza A(H5Nx) clades 2.3.4.4b and 2.3.2.1c in humans

December 14, 2023

Important dates:

Evidence published up to December 14, 2023

Protocol initiated December 14, 2023

Protocol finalized January 5, 2024

# **Background**

The avian influenza A(H5Nx) clade 2.3.4.4b viruses first emerged between 2018-2020, spreading globally to become the predominant clade in both wild birds and domestic poultry in Asia, Africa, Europe, and the Middle East. The A(H5N1) clade 2.3.4.4b virus emerged in late 2020 and quickly outpaced the previously circulating A(H5N6) and A(H5N8) viruses by spring 2021, and has continued to spread globally ever since (Centers for Disease Control and Prevention, 2023). The Canadian Food Inspection Agency (CFIA) first confirmed the A(H5N1) clade 2.3.4.4b virus in Canada in December 2021 in Newfoundland and Labrador. Since then, almost 11 million birds from commercial and non-commercial poultry flocks in Canada have been culled or have died from infection with the virus. A(H5N1) has impacted an extraordinary number of wild bird species around the world as well, leading to reports of large die-offs, as well as concerns for at-risk populations. Avian influenza A(H5N1) viral activity has been unprecedented in terms of the number of animal species infected, the extensive geographic spread, and duration of the epizootic.

Since October 2020, avian influenza A(H5N1) and A(H5) infections in mammals have been reported in Europe, Asia and the Americas, affecting numerous species of wild and domestic mammals, as well as marine mammals (Centers for Disease Control and Prevention, 2023). In Canada, wild mammal species reported to have been infected with A(H5N1) include, but are not limited to, foxes, skunks, raccoons, bears, seals and dolphins. Avian influenza A(H5N1) has also been reported in a domestic dog and several domestic cats in Canada, as well as domestic cats in Europe and Asia, and farmed fur animals in Europe. Large numbers of marine mammals have been infected with A(H5N1) during the current global epizootic, including seals, sea lions, elephant seals as far south as Argentina.

The clade 2.3.2.1c is another clade of the A(H5N1) virus that has been predominantly circulating in poultry in Cambodia, Laos and South America. Over the past years this clade has only be detected in Asia

EPIDEMIOLOGICAL SUMMARY OF THE 19 RECENT HUMAN CASES OF H5N1

The illness onset dates for the **six clade 2.3.2.1C** A(H5N1) cases range from February 23, 2023, to November 25, 2023. The median age of these cases was 16 years (range: 2-59) and one-third (2/6) of the cases were male. All of these cases were reported from Cambodia, from three different regions: Prey Veng Province (3), Kampot Province (2), and Svay Reing Province (1). Only one (1/6) of the A(H5N1) clade 2.3.2.1c cases experienced mild illness. **The other cases (5/6) were all severe in nature, with 4 reported fatalities** (4/6) (Public Health Agency of Canada, 2023). All of the A(H5N1) clade 2.3.2.1c cases reported exposure to birds prior to illness onset.

The illness onset dates for the **13 clade 2.3.4.4b** A(H5N1) cases range from January 6, 2022, to July 14, 2023 (Public Health Agency of Canada, 2023). Specific ages were available for eight of the A(H5N1) clade 2.3.4.4b cases, which revealed a median age of 33 years (range: 5-80). Sex was available for 12, eight of whom were male. These cases were reported from the seven other countries: England (5), Spain (2), China (2), the United States (1), Ecuador (1), Chile (1), and Vietnam (1). **Most (8/13) of the cases experienced either mild illness or were asymptomatic**. Five (5/13) were severe in clinical presentation, with one fatality reported in one of the A(H5N1) clade 2.3.4.4b cases from China. Other than one case that reported exposure to a contaminated environment (case from Chile), the rest of these cases (12/13) reported exposure to poultry prior to illness onset.

## Rationale

At the request of senior management, for a comprehensive summary of what is known, a rapid review (RR) will be conducted contrasting avian influenza clade 2.3.2.1c and clade 2.3.4.4b in humans. A variety of virological and epidemiological outcomes will be contrasted to identify similarities and differences. See detailed questions and definitions below.

# **Methods**

## Team

| **Affiliation** | **Collaborators** | **Expertise** | **Role** |
| --- | --- | --- | --- |
| PHRS- SOR-NMLB- PHAC | Dr. Lisa Waddell  Tricia Corrin (lead)  Kaitlin Young  Mavra Qamar  Kusala Pussegoda  Austyn Baumeister | Synthesis research expertise | - Conduct all stages of rapid review - Draft paper |
| IERIS-CERIPP-IDVPB- PHAC | Dr. Nicole Atchessi  Simran Sandhu  Alex Vasiliu  Christina Ferrante | Avian influenza expertise | - Participate in the conduct all stages of rapid review - Provide expert guidance - Provide edits to paper |
| ZD-CFEZID-IDVPB-PHAC | Dr. Erin Leonard  Dr. Danielle Julien | Avian influenza expertise | - Provide expert guidance - Provide edits to paper |

## Research questions

1. Is there a difference in animal-to-human transmissibility between avian influenza clade 2.3.4.4b and 2.3.2.1c?
2. Do the epidemiological characteristics of human cases differ between avian influenza clade 2.3.4.4b and 2.3.2.1c?
3. For avian influenza clade 2.3.4.4b and 2.3.2.1c in humans are there differences in the adaptation, transmissibility, pathogenicity or virulence?

**Pathogens:** Avian influenza clade 2.3.4.4b or 2.3.2.1c, all hemagglutinin (H 1-16) and neuraminidase (N 1-9) will be screened into the project and coded in the evidence map.

**Population:** Question 1, 2 and 3 includes all humans. Question 1 may include any animals, mammals or birds.

**Outcomes:** A range of outcomes are expected to underpin the different questions. The following is a list of outcome categories and definitions to be used in mapping the literature.

- **Zoonotic transmission** is the transmission of the pathogen between animals and humans. Data may come from *in silico* or *in vitro* experiments, or a range of descriptive and analytical epidemiological investigations.
- **Other potential modes of transmission between humans or animals** given avian influenza is a respiratory virus, there are four major [modes of transmission](https://www.nature.com/articles/s41579-021-00535-6): direct (physical) contact, indirect contact (fomite), (large) droplets and (fine) aerosols. Little is known about the relative contribution of each mode to the transmission of a particular virus in different settings.
- **Risk factors for exposure** from descriptive and analytical epidemiological investigations we can extract evidence on situations that result in increased or decreased risk of exposure.
- **Risk factors for severe disease/mortality** from descriptive and analytical epidemiological investigations are often conducted in a clinical setting for humans but may also be field investigations (for both animals and humans) that examine associations of individual attributes with the risk of severe disease or mortality.
- **Description of clinical disease** from descriptive and analytical epidemiological investigations. These studies are often reporting on the frequency of symptoms and specific pathologies of disease in a sample that ideal represents the spectrum of disease in a target population.
- **Adaptation** is a change or the process of change by which an organism or species becomes better suited to its environment. In this RR we will compare and contrast the evidence for adaptation to animals or humans across the two clades. Examples of topics that fall within this topic:
  - Acquisition of certain mutations known to (or shown to via an *in vitro* or *in vivo* experiment) signal mammal or human adaptation. E.g. mutations that enhance attachment to mammalian cells, mutations that lead to enhanced replication,
  - Epidemiological evidence, often surveillance of circulating subtypes overtime, of improved viral fitness (improved transmission, virulence etc). e.g. a reassortment occurs and a new subtype A(HxNx) subtype becomes dominant.
  - Animal model testing of new subtypes vs. old subtypes shows increased transmission/pathogenicity and/or virulence. (May include an explanation of the mutations thought to contribute to this improved fitness).
  - NOT RELEVANT: Are studies that only discuss / demonstrate the increased risk of new avian influenza subtypes with multiple subtypes are circulating and there is evidence of on-going introductions (often from migratory birds). We are looking for the evidence of this occurring rather than just an acknowledgement of the situations under which there is increased pressure.
- **Transmissibility** is a measure of how easily a pathogen spreads from an infected to a susceptible. We typically measure this by basic reproduction number (*R*_0_) or secondary attack rate derived from modelling studies or observational studies. However, animal models, volunteer transmission studies, and interventional epidemiological studies can also contribute to what we know on [transmissibility.](https://www.nature.com/articles/s41579-021-00535-6)
  - **Determinants of transmission**
    - The infectivity of the pathogen,
    - How contagious an infected is (how much virus is shed),
    - The susceptibility of an exposed (combination of existing immunity and level of exposure),
    - The environmental stress on the pathogen during transmission (survival of pathogen).
- **Pathogenicity** is the capacity of a pathogen to cause damage in a host (i.e., harm the host). This ability represents a genetic component of the pathogen and the overt damage done to the host is a property of the host-pathogen interactions. This is considered binary – pathogenic or not to a host.
- **Virulence** is the degree of damage caused by a pathogen. The extent of the virulence is usually correlated with the ability of the pathogen to multiply within the host and may be affected by other factors (i.e., the disease producing power of an organism and the degree of pathogenicity).

## Eligibility criteria

All citations identified by the search strategy will be assessed for inclusion in the RR according to the eligibility criteria presented below.

Include

- English and French articles
- Primary research
- Avian influenza clades 2.3.4.4b and 2.3.2.1c
- Articles related to AIV in humans and human exposure to AIV.

Exclude

- Citations published in other languages at full text so we can characterize language bias
- Secondary (literature reviews) or tertiary (books) citations, commentaries/editorials/opinions etc. that do not present data collected by the author.
- Other pathogens and avian influenza clades
- Articles that do not address one of the foci presented in the questions above (e.g., prevalence, spread in animals, therapeutics/other interventions in all populations)

## Search

The electronic search will be conducted in PubMed and Scopus and EuropePMC and includes searching for and identification of preprints (all three index preprints, EuropePMC has been validated as up to date and accurate). The search has no date limits or language limits. The search results will be managed and deduplicated in Refworks. Another round of deduplication will be done in DistillerSR.

**Avian Influenza clade 2.3.4.4b**

**Pubmed**

((((influenza A virus[MeSH Terms]) OR ("influenza in birds"[MeSH Terms]) OR (avian influenza[Title/Abstract]))) AND ("2 3 4 4b"[Title/Abstract] OR "2 3 4 4b 2"[Title/Abstract] OR "2 3 4 4b a"[Title/Abstract] OR "2 3 4 4b a h5n8"[Title/Abstract] OR "2 3 4 4b avian"[Title/Abstract] OR "2 3 4 4b avian influenza"[Title/Abstract] OR "2 3 4 4b avian influenza virus"[Title/Abstract] OR "2 3 4 4b clade"[Title/Abstract] OR "2 3 4 4b h5n1"[Title/Abstract] OR "2 3 4 4b h5n1 hpaiv"[Title/Abstract] OR "2 3 4 4b h5n6"[Title/Abstract] OR "2 3 4 4b h5n8"[Title/Abstract] OR "2 3 4 4b molecular"[Title/Abstract] OR "2 3 4 4b molecular detection"[Title/Abstract] OR "2 3 4 4b reassortant"[Title/Abstract]))

**EuropePMC** – publications and preprints

DISEASE:influenza AND (2.3.4.4b OR "2 3 4 4b" OR "2 3 4 4 b")

**Scopus**

TITLE-ABS-KEY ((“influenza A virus”) OR ("influenza in birds") OR (“avian influenza”)) AND TITLE-ABS-KEY ((“2.3.4.4b”) OR ("2 3 4 4b") OR ("2 3 4 4b 2") OR ("2 3 4 4b a") OR ("2 3 4 4b a h5n8") OR ("2 3 4 4b avian") OR ("2 3 4 4b avian influenza") OR ("2 3 4 4b avian influenza virus") OR ("2 3 4 4b clade") OR ("2 3 4 4b h5n1") OR ("2 3 4 4b h5n1 hpaiv") OR ("2 3 4 4b h5n6") OR ("2 3 4 4b h5n8") OR ("2 3 4 4b molecular") OR ("2 3 4 4b molecular detection") OR ("2 3 4 4b reassortant"))

___________________________________________________________________

**Avian Influenza clade 2.3.2.1c**

**Pubmed**

(((influenza a virus[MeSH Terms]) OR (influenza in birds[MeSH Terms])) OR (avian influenza[Title/Abstract]))) AND ("2.3.2.1c"[Title/Abstract] OR "2 3 2 1c"[Title/Abstract] OR "2 3 2 1c eurasian"[Title/Abstract] OR "2 3 2 1c eurasian lineage"[Title/Abstract] OR "2 3 2 1c h5n1"[Title/Abstract] OR "2 3 2 1c h5n1 virus"[Title/Abstract])

**EuropePMC** – publications and preprints

DISEASE:influenza AND (2.3.2.1c OR "2 3 2 1c" OR "2 3 2 1 c")

**Scopus**

TITLE-ABS-KEY(("influenza a virus") OR ("influenza in birds") OR ("avian influenza")) AND TITLE-ABS-KEY ( ( "2 3 2 1c" ) OR ( "2 3 2 1c eurasian" ) OR ( "2 3 2 1c eurasian lineage" ) OR ( "2 3 2 1c h5n1" ) OR ( "2 3 2 1c h5n1 virus" ) OR ( "2.3.2.1c" ))

## Search results

| **Search date** | **Clade** | **Pubmed** | **Scopus** | **EuropePMC** |
| --- | --- | --- | --- | --- |
| Dec 14, 2023 | 2.3.4.4b | 232^†^ | 243 | 420 |
| Dec 14, 2023 | 2.3.2.1c | 69 | 72 | 209 |

^†^only 20 of the hits are pre2020

## Search verification and grey literature search

Four relevant systematic reviews were identified by the electronic search and were used for search verification (Chauhan, Dessie, Noreddin, & El Zowalaty, 2020; Chauhan & Gordon, 2022; Kalonda et al., 2021; Tahmo et al., 2023), the reference lists were screened for relevant articles omitted by our search. No citations were added to the review from this screening.

To identify the most recent case and outbreak reports the websites of the world health organization, Food and agricultural organization, European food safety authority, the US Centers for Disease Control and Prevention, the Pan American Health Organization and the UK Health Security Agency avian influenza webpages were examined for recent reports. Five reports (Centers for Disease Control and Prevention, 2023; European Food et al., 2023; Food and Agricultural Organization of the United Nations, 2023; Pan American Health Organization & World Health Organization, 2023; World Health Organization, 2024) with reference lists were identified and screened for relevant citations that were omitted by the electronic search. From this 1 technical report (UK Health Security Agency, 2023) and 4 outbreak reports (World Health Organization, 2023a; World Health Organization, 2023b; World Health Organization, 2023c; World Health Organization, 2023d) were added to the rapid review for screening and potential inclusion. Based on the search verification, all potentially relevant published research had been identified by the electronic search.

## Review management

The RIS files from the search will be imported into DistillerSR, a web-based systematic review software designed to manage all stages of conducting synthesis research. All stages of the RR including deduplication, relevance screening, and data extraction will be conducted in DistillerSR. The final dataset will be exported into MS Excel, cleaned and tabulated for use in the final product.

## Relevance screening – full text

Two reviewers will screen full text independently for relevance based on eligibility criteria. Conflicts that cannot be resolved will be reconciled by a third reviewer.

Full text screening tool

| **Question** | **Options** | **Definitions/additional notes** |
| --- | --- | --- |
| **Is this article on avian influenza clade 2.3.4.4b or 2.3.2.1c?**  Specify the hemagglutinin (H1-16) and neuraminidase (N1-9) nomenclature for the avian influenza **isolated or** **studied** in this article.  *Check all that apply and add as needed* | - Yes - **2.3.4.4b**   - - N/A     - H5N1       - Fully Eurasian       - Reassortment with N. American lineages       - Details unknown     - H5N6     - Add as needed - **2.3.2.1c**   - - N/A     - H5N1     - H5N6     - Add as needed - No (exclude) | Within the clades of interest there different haemagglutinin and neuraminidase compositions that have been studied. Indicate the HxNx of the avian influenza in the article unless it's a phylogenetic analysis of many genomes.  NOTE: 2.3.4.4b H5N1 underwent a reassortment in North America in 2021 after which as there have been changes in epidemiology, spread and pathogenicity. Specify if the study was on the fully Eurasian strain (before reassortment) or on the (reassortment strain ~ 2021 onward was circulating).  **Select N/A** when its a review or the authors did not collect the samples (e.g. in a phylogenetic analysis), focus on categorizing the AI identified / studied.  Avoid adding **HxNx** where they did not confirm what virus they have in the sample – not helpful. |
| What language is the article published in? | - English - French - Other, please specify [TXT]   (exclude) | Relevance verification questions should be answered for all languages. |
| Does this article describe primary research? | - Yes – primary research - Peer-reviewed journal article - Preprint - PhD/MSc thesis - Report - Conference paper - Letter to the editor - Short communication - Commentary - Study protocol - Other, please specify: [TXT] - No – non-primary research - Systematic review - Scoping review - Rapid review or other evidence synthesis - Narrative Review - Conference proceeding - Book - Other non-primary sources (letter to the editor, commentary, news etc.) | **Primary research** represents a study where the authors collected and analysed their own data.  We will include journal articles, PhD/MSc Theses, reports, conference papers, and letters to the editor, short communications, and commentaries **if they contain primary data**.  **Review** is a comprehensive or brief narrative or systematic review summarizing knowledge on an issue. *Please specify SR, ScR or other evidence synthesis if applicable.*  Other **non-primary** literature will encompass letters to the editor, commentaries, opinion pieces, etc. |
| What question(s) does this article address?  *Check all that apply* | - The study is on animal-to-human transmissibility for avian influenza clade 2.3.4.4b or 2.3.2.1c - This study describes the epidemiological characteristics of human cases of avian influenza clade 2.3.4.4b or 2.3.2.1c - This study describes avian influenza clade 2.3.4.4b or 2.3.2.1c **in humans** and has outcomes related to adaptation, transmissibility, pathogenicity, or virulence - This study describes avian influenza clade 2.3.4.4b or 2.3.2.1c **in animals** and has outcomes related to adaptation, transmissibility, pathogenicity or virulence (EXCLUDE) - Does not address any of the research questions (EXCLUDE) | **Adaptation** is a change or the process of change by which an organism or species becomes better suited to its environment.  -This could be about the acquisition of certain mutations and epidemiological evidence of improved viral fitness (improved transmission, virulence etc.)  **Transmissibility** is a measure of how easily a pathogen spreads from an infected to a susceptible. We typically measure this by basic reproduction number (*R*_0_) or secondary attack rate derived from modelling studies or observational studies.  **Pathogenicity** is the capacity of a pathogen to cause damage in a host (i.e., harm the host). This ability represents a genetic component of the pathogen and the overt damage done to the host is a property of the host-pathogen interactions. This is considered binary – pathogenic or not to a host.  **Virulence** is the degree of damage caused by a pathogen. The extent of the virulence is usually correlated with the ability of the pathogen to multiply within the host and may be affected by other factors (i.e., the disease producing power of an organism and the degree of pathogenicity). |
| Additional comments | [TEXT] |  |

## Data extraction

The data extraction form will be completed by a single reviewer. This will be verified by a senior reviewer and the senior reviewer will establish if the included study can be assessed for risk of bias based on the study design. Any discrepancies will be resolved by consensus or a third reviewer where necessary.

Data extraction tool

| **Question** | **Options** | **Definitions/additional notes** |
| --- | --- | --- |
| In what country was the study conducted?  *Check all that apply* | - Canada - United States - *Add as needed* | This should be the country where the samples are collected.   - You may use author affiliation to help determine a country of study conduct for experiments. - For phylogenetic analyses, please determine where the analysis was conducted rather than where the genomes came from. |
| When was the study (samples collected) conducted? Answer format MM-YYYY to MM-YYYY | *Txt* MM-YYYY to MM-YYYY | This should be the range of when the samples are collected.  But if this is a laboratory study, we may only have date of first submission of the study in which case indicate **EST MM-YYYY** |
| What is the study design? | - Observational study - Case control - Cross-sectional - Retrospective cohort - Prospective cohort - Case report/series - Longitudinal - Surveillance or monitoring program - Ecological study - Other OBS: ___ - Experimental study - Controlled Trial - Challenge trial - Other EXP:___ - Descriptive studies - Cluster investigation - Outbreak investigation - Prevalence study - Qualitative research - Biological monitoring studies - *In vitro* - *In silico*   - Genetic sequencing   - Phylogenetic analysis - Evaluation of a diagnostic test - Predictive Model - Risk Assessment   Other: specify ____ | **Observational study**: Detection or surveillance of pathogens in humans and animals is conducted without manipulation or intervention.  **Case control**: Usually retrospective, identified cases are matched with controls and predictors of disease are identified. In this review, we may see studies that use different communities as cases and controls.  **Cross-sectional**: Examines the relationship of risk factors and outcome at a point in time on a representative sample of the target population.  **Cohort**: follow a group of exposed and non-exposed individuals to evaluate whether they develop an outcome or retrospectively evaluate exposure/disease when the exposure was likely to be a point source.  **Case series/report**: A single person or collection of people/patients with common characteristics used to describe some clinical, risk of exposure or operational aspect of a disease, treatment, etc.  **Longitudinal studies**: This is similar to a **prevalence study** but instead of being done at one point in time, it is done repeatedly. It is different from a cohort study in that it is not usually the same individuals who are studied at each time point  **Surveillance/monitoring program results**: On-going sampling from a defined representative sample of the target population to evaluate changes over time.  **Ecological study:** The study of an association between an exposure and outcome when the units of analysis are populations or groups of people (aggregate) rather than individuals  **Experimental study**: Researchers manipulate one or more variables during the course of the study.  **Controlled trial**: experiments where the investigator has control over the experiment. Typically involves the comparison of a control group against an experimental group that is identical in all aspects except the exposure of interest.  **Challenge trial** is a controlled trial that includes exposure of a human or animal to the pathogen.  **Other** may include controlled before and after study and interrupted time-series study which we are not expecting to have in this study.  **Descriptive studies:**  **In silico study**: in silico is an expression used to mean “performed on computer or via computer simulation”. There is a variety of in silico techniques, but the three that are discussed most are:  **Genetic sequencing:** as an alternative to *in vitro*methods for identifying bacteria, various *in silico* methods which sequence bacterial DNA have been developed.  **Phylogenetic analysis:**  Study of the evolutionary development of a species or a group of organisms.  **Qualitative Study:** The study of social phenomena to answering questions of “why” and “how” through focus groups, interviews, surveys, self-reports, observations, or document analysis.  **Diagnostic Test Accuracy** study investigates the performance of a test to diagnose disease or a condition.  **Predictive model**: Predictive modeling is a mathematical process used to predict/forecast future events or outcomes.  **Risk assessment:** determines the quantitative or qualitative value of risk related to a situation or recognized threat. Quantitative risk assessment requires calculation of the magnitude of the potential loss and the probability that the loss will occur. |
| **What is the source of funding for this study?** | - Pharmaceutical company - Other (gov, uni, hospital etc.) specify _____ - None - Not reported |  |
| **Provide conflicts of interest and any justifications that the study was done independently of the funding sources.** | - TxT   If this information is not reported, put "N/A" |  |
| What type of animals or humans were sampled in this study? | - Humans   - Txt: specify if a notable subgroup. E.g. pediatric, occupational, elderly etc. - Mammals - Wild   - - Add options as they occur - Domestic   - - Add options as they occur - Birds - Wild   - - Add options as they occur - Domestic   - - Add options as they occur | Please use the scientific name for all mammals and birds |
| Methods: describe the population included in the sampling frame or included in the experiment. | - txt | WHO IS INCLUDED IN THE STUDY?  Include key characteristics if relevant related to when/where/ demographics/ subpopulations sampled etc.  Or related to balancing confounders in the study groups. |
| Methods: setting of the study | - laboratory/controlled environment - Field samples - TxT | WHERE WAS THE STUDY CONDUCTED?  Establish the circumstances around obtaining the study samples, these are very controlled for experiments, but for observational studies are highly variable, so describe the where, when and how it was decided to take a sample (e.g. consecutive, convenience, etc.) |
| Methods: briefly indicate the methods for detection and characterization of the virus in the study | - txt | e.g. RT-PCR, WGS, antigenic testing, serology etc. |
| Methods: Briefly describe the methods of analysis used in the study | - a phylogenetic analysis is included in this study. - txt | e.g. descriptive, regression analysis, phylogenetic analysis, etc. |
| Additional comments on this study? | - Txt |  |
| **For DE verifier**: Should risk of bias assessment be completed?  What tool is appropriate? | - Yes   - Newcastle-Ottawa Scale for case-control studies   - Newcastle-Ottawa Scale for cohort studies   - Adapted version of the Newcastle-Ottawa Scale for cross-sectional studies   - Quality Assessment of Diagnostic Accuracy Studies 2 (QUADAS-2)   - JBI Critical Appraisal Checklist for prevalence studies   - JBI Critical Appraisal Checklist for case reports   - JBI Critical Appraisal Checklist for case series   - ROB 2.0   - ROBINS-I - No | This is to be completed once DE verification has been done by the senior reviewer. |
| Repeating Form | ONE OUTCOME PER FORM! |  |
| Clade for this line of data | - 2.3.4.4b *txt specify the NxHx*____________ - 2.3.2.1c *txt specify the NxHx*____________ |  |
| Select the outcome assessed in this line of data (NOTE: 1 outcome per line) | - Transmissibility of virus from animal to human - zoonotic (Q1) - Clinical data on human cases (Q2) - Epidemiological data on human cases (Q2) - Adaptation in humans (Q4) - Transmissibility in humans (Q4) - Pathogenicity and/or virulence in humans (Q4) - Adaptation in animals (Q3) - Transmissibility in animals (Q3) - Pathogenicity and/or virulence in animals (Q3) | See definitions in the protocol.  ONLY extract data that is relevant to one of our questions. We do not want any lines of data that are not relevant.  On the main DE form there is a comment box to note anything including other available outcomes in the paper if you feel it is important. |
| Population represented in this line of data | TXT_____________________________ | Describe who/what was sampled. Note animal species, human, and any subpopulation information applicable to this line of data or if there is a control group / exposed and unexposed group etc.  e.g. this sample is of people who live in households where chickens recently died. |
| Total N in this line of data | TXT_____________________________ | Indicate the total number of observations in this line of data. (and/or the number in each group.) e.g. if this is an experiment there may be an intervention group n=4 mice and a control group n=4 mice. |
| What was the confirmation of avian influenza status for this line of data. | TXT______________________________ | Methods have been extracted in the main form. In this box indicate if this was disease confirmed by PCR and neutralization test or serology etc. so we can link to the methods. |
| Intervention (if applicable) assessed in this line of data | TXT_____________________________ | Describe the intervention, timing, dose etc. |
| Risk Factor (if applicable) assessed in this line of data | TXT_____________________________ | Describe the risk factor being measured and determination or confirmation of the risk factor. |
| Numerical Results:   - Prevalence: +/n or % and n. - Measure of central tendency: indicate the measurements (e.g. mean, median) and dispersion (SE, SD, 95%CI) by group. - Measure of effect: indicate measure e.g. MD and dispersion (E.g. SE, SD, 95% CI) and p-value - Measure of Association: e.g. RR, OR, HR, IRR, PR and 95%CI, p-value. - Other statistic: indicate what it is, the statistics, measure of dispersion and p-value. | TXT_____________________________ | Note: if the outcome is reported form a univariate (unadjusted) and multivariate (adjusted) analysis… report both and be clear about which adjusted.  e.g. OR 2.3 (95%CI 1.4- 4.7) adjusted, p-value 0.02.  Standardized mean difference 0.36 ( SE 0.29), p-value 0.04. |
| Was the numerical result adjusted? | - Yes (list what it was adjusted for) TxT________ - No - Not reported | If there is a multivariable model, please list what the estimate in the above box was adjusted for. E.g. Estimates and odds ratios are adjusted for age, sex, white or non-white ethnicity. |
| Please interpret the numerical result so the direction of the effect or association is easily understood: | TXT_____________________________ | e.g. A poultry worker has a 4 times higher odds of being seropositive for H5N1 compared to the general population. |
| non-numerical results for this line of data: | TXT_____________________________ | This is ONLY the results related to this line of data. Be concise and direct. We do not want to wade through a bunch of text. |
| Mutations of significance: | Check of the mutation:   - Add as they occur   TXT_____________________________ | These would be mutations presented by the author as conferring some advantage or disadvantage. Ideally with an explanation as to what the impact of the mutation is e.g. confers better binding affinity to mammalian cells than a predecessor clade or other strain (make sure this is specified.) |
| Please note the following about this line of data: | TXT_____________________________ | Optional Textbox in case there is some other information that is important to this line of data. |

## Risk of Bias

While data will be extracted for all study designs, quality assessment will only be conducted for study designs that have validated tools available. This includes the following study designs: case-control, cohort, cross-sectional, diagnostic test accuracy studies, surveillance data analyses, prevalence studies, case reports and case series. Quality assessment will be performed by one reviewer and verified by a second senior reviewer. The full risk of bias tools used can be found in Appendix 1.

The quality assessment tools that will be used where applicable include:

- Newcastle-Ottawa Scale for case-control studies evaluates the risk of bias associated with selection, comparability and exposure in the study and has a rating scale for risk of bias (Wells, O'Connell, Robertson, Welch, & Tugwell, 2021).
  - - Out of a potential 9 points across 3 domains, a score of 0-5 = high risk of bias, 6-7 = medium risk of bias, and 8-9 = low risk of bias
- Newcastle-Ottawa Scale for cohort studies evaluates the risk of bias associated with selection, comparability and outcome in the study and has a rating scale for risk of bias (Wells, O'Connell, Robertson, Welch, & Tugwell, 2021).
  - - Out of a potential 9 points across 3 domains, a score of 0-5 = high risk of bias, 6-7 = medium risk of bias, and 8-9 = low risk of bias
- Adapted version of the Newcastle-Ottawa Scale for cross-sectional studies evaluates the risk of bias associated with selection, comparability and outcome in the study and has a rating scale for risk of bias (Herzog et al., 2013).
  - - Out of a potential 9 points across 3 domains, a score of 0-5 = high risk of bias, 6-7 = medium risk of bias, and 8-9 = low risk of bias
- JBI Critical Appraisal Checklist for prevalence studies is a 9 domain questionnaire that evaluates the bias and complete reporting (Munn, Moola, Lisy, Riitano, & Tufanaru, 2015).
- JBI Critical Appraisal Checklist for case reports is an 8 domain questionnaire that evaluates the complete reporting of cases, yes indicates the item was reported (Moola et al., 2020).
- JBI Critical Appraisal Checklist for case series is a 10 domain questionnaire that evaluates the complete reporting of case series, yes indicates the item was reported (Munn et al., 2020).

Validated quality assessment tools are not available for the following study designs: cluster investigations, in vitro, in silico, phylogenetic analyses, exposure investigations, and mathematical and predictive epidemiological models. Therefore, quality assessment will not be performed for articles with these study designs.

## Data analysis

Data analysis will largely be narrative synthesis as there are likely few quantitative outcomes to extract. Studies will be grouped for analysis within the question they are addressing and within that they will be grouped by sub-topic e.g. transmissibility, adaptation or pathogenesis and virulence. Within these sub-groups data will be grouped by outcome and species.

Numerical outcomes of association will ideally be converted to odds ratios for comparison across studies. Prevalence outcomes ideally are reported with the number positive, denominator and proportion. A measure of variability should be reported when available. Additional numerical outcomes have not been anticipated *a priori* but will be added below if identified.

If there are numerical outcomes and two or more studies considered to be comparable and appropriate for a meta-analysis. A random effects meta-analysis will be conducted using an appropriate weighting procedure depending on the outcome measure. This will be conducted in STATA 18, and will include production of a forest plot and prediction intervals. Heterogeneity will be evaluated using the statistics produced by the meta-analysis. The funnel plot can be examined for evidence of publication bias and if there are more than 10 lines of data Egger’s, Begg’s and Duval and Tweedie’s trim and fill methods can also be used to evaluate potential publication bias.

When synthesis does not include a meta-analysis, the narrative description will include the number of studies contributing to the outcome group, the type of research, and an evaluation of the consistency of that research across studies and risk of bias (if applicable).

It is not expected that there will be a lot of research comparing the two clades 2.3.4.4b and 2.3.2.1c directly. If there is head to head studies these will be highlighted as important contributions to the question. The synthesized results by clade will then be contrasted across clades 2.3.4.4b and 2.3.2.1c to answer the research questions one sub-question at a time. It will be important to provide the amount and type of research, an evaluation of consistency and uncertainty in the findings, as well as a reflection on the knowledge gaps that persist.

## Appendix 1. Risk of bias tools

### RoB scoring matrix.

| **Study Design** | **RoB Rating** | **Score** |
| --- | --- | --- |
| **Cohort and Case-Control** | Low RoB | 8-9 points |
|  | Medium RoB | 6-7 points |
|  | High RoB | 0-5 points |
| **Cross-Sectional** | Low RoB | 7-8 points |
|  | Medium RoB | 6 points |
|  | High RoB | 0-5 points |

### Newcastle Ottawa RoB rating tools.

| **Question** | **Options** |
| --- | --- |
| **Cohort Studies** | |
| **Selection (maximum 4 points)** | |
| Representativeness of the exposed cohort | - Truly representative of the average in the community (**1 POINT) [TEXT] - Somewhat representative of the average in the community [TEXT] - Selected group of users (e.g. nurses, volunteers) [TEXT] - No description of the derivation of the cohort [TEXT] |
| Selection of the non-exposed cohort | - Drawn from the same community as the exposed cohort (**1 POINT) [TEXT] - Drawn from a different source [TEXT] - No description of the derivation of the non-exposed cohort [TEXT] |
| Ascertainment of exposure | - Secure record (e.g. hospital records) (**1 POINT) [TEXT] - Structured interview [TEXT] - Written self-report [TEXT] - No description [TEXT] |
| Demonstration that outcome of interest was not present at start of study | - Yes (**1 POINT) [TEXT] - No [TEXT] |
| **Comparability (maximum 2 points)** | |
| Subjects in different outcome groups are comparable, based on study design or analysis. Confounding factors are controlled. | - Study controls for age and sex (**1 POINT) [TEXT] - Study controls for other factors such as comorbidities and socioeconomic status (**1 POINT) [TEXT] - Not reported or no control [TEXT] |
| **Outcome (maximum 3 points)** | |
| **Case-Control Studies** | |
| **Selection (maximum 4 points)** | |
| Is the case definition adequate? | - Yes, with independent validation (e.g. >1 person/record/time/process to extract information) (**1 POINT) [TEXT] - Yes (e.g. medical records such as ICD codes in database or based on self reports with no reference to a primary record) [TEXT] - No description of how a case is defined [TEXT] |
| Are the cases representative? | - Consecutive or obviously representative series of cases (**1 POINT) [TEXT] - Potential for selection biases [TEXT] - Not stated [TEXT] |
| Are the selection of controls from the same community? | - Community controls (i.e. same community as cases and would be cases if they had the outcome) (**1 POINT) [TEXT] - Hospital controls (i.e. within same community as cases but derived from a hospitalized population) [TEXT] - Controls were selected from another population or no description of how controls were selected [TEXT] |
| Definition of controls | - Controls have no history of the outcome/disease/endpoint. If cases are first occurrence of the outcome of interest, then it must explicitly state that controls have no history of this outcome. If cases have new (not necessarily first) occurrence of outcome, then controls with previous occurrences of outcome of interest should not be excluded. (**1 POINT) [TEXT] - No mention of history of outcome/disease/endpoint for the controls [TEXT] |
| **Comparability (maximum 2 points)** | |
| Comparability of cases and controls on the basis of the design or analysis | - Study controls for_(most important factor) - Study controls for any additional factor (This criteria could be modified to indicate specific control for a second important factor.) |
| **Exposure (maximum 3 points)** | |
| Ascertainment of exposure | - Secure record (e.g. surgical records) (**1 POINT) [TEXT] - Structured interview where blind to case/control status (**1 POINT) [TEXT] - Interview not blinded to case/control status [TEXT] - Written self-report or medical record only [TEXT] - No description [TEXT] |
| Same method of ascertainment for cases and controls | - Yes (**1 POINT) [TEXT] - No [TEXT] |
| Non-response rate | - Same rate for both groups (**1 POINT) [TEXT] - Non-respondents described [TEXT] - Non-response rate different and no designation [TEXT] |
| RoB Score | - Low (8-9 points) [TEXT] - Medium (6-7 points) [TEXT] - High (0-5 points) [TEXT] |
| **Cross-Sectional Studies** | |
| **Selection (maximum 3 points)** | |
| Representativeness of the sample | - Truly representative of the average in the community (**1 POINT) [TEXT] - Somewhat representative of the average in the community [TEXT] - Selected group of users (e.g. nurses, volunteers) [TEXT] - No description [TEXT] |
| Sample size | - Sample size justified and satisfactory (**1 POINT) [TEXT] - Sample size not justified or reported [TEXT] |
| Non-included subjects | - Comparability between included and non-included subject established and satisfactory (**1 POINT) [TEXT] - Comparability between included and non-included subjects not established and satisfactory [TEXT] |
| **Comparability (maximum 2 points)** | |
| Subjects in different outcome groups are comparable, based on study design or analysis. Confounding factors are controlled. | - Study controls for age and sex (**1 POINT) [TEXT] - Study controls for other factors such as comorbidities and socioeconomic status (**1 POINT) [TEXT] - Not reported or no controls [TEXT] |
| **Outcome (maximum 3 points)** | |
| Assessment of outcome | - Independent blind assessment (**1 POINT) [TEXT] - Medical records [TEXT] - Self-report (**1 POINT) [TEXT] - No description [TEXT] |
| Statistical test | - Statistical test used was clearly described and appropriate, and the measurement of the association was presented with CI's and p-value (**1 POINT) [TEXT] - Statistical test was not clearly described or appropriate [TEXT] |

### Joanna Briggs Institute Critical Appraisal Checklists.

| **Question** | **Options** |
| --- | --- |
| **Prevalence Studies** | |
| Was the sample frame appropriate to address the target population? | - Yes - No - Unclear - Not applicable |
| Were study participants sampled in an appropriate way? | - Yes - No - Unclear - Not applicable |
| Were the study subjects and the setting described in detail? | - Yes - No - Unclear - Not applicable |
| Was the data analysis conducted with sufficient coverage of the identified sample? | - Yes - No - Unclear - Not applicable |
| Were valid methods used for the identification of the  condition? | - Yes - No - Unclear - Not applicable |
| Was the condition measured in a standard, reliable way  for all participants? | - Yes - No - Unclear - Not applicable |
| Was there appropriate statistical analysis? | - Yes - No - Unclear - Not applicable |
| Was the response rate adequate, and if not, was the low response rate managed appropriately? | - Yes - No - Unclear - Not applicable |
| **Case Reports** | |
| Were patient’s demographic characteristics clearly described? | - Yes - No - Unclear - Not applicable |
| Was the patient’s history clearly described and presented as a timeline? | - Yes - No - Unclear - Not applicable |
| Was the current clinical condition of the patient on presentation clearly described? | - Yes - No - Unclear - Not applicable |
| Were diagnostic tests or assessment methods and the results clearly described? | - Yes - No - Unclear - Not applicable |
| Was the intervention(s) or treatment procedure(s) clearly described? | - Yes - No - Unclear - Not applicable |
| Was the post-intervention clinical condition clearly described? | - Yes - No - Unclear - Not applicable |
| Were adverse events (harms) or unanticipated events identified and described? | - Yes - No - Unclear - Not applicable |
| Does the case report provide takeaway lessons? | - Yes - No - Unclear - Not applicable |
| **Case series** | |
| Were there clear criteria for inclusion in the case series? | - Yes - No - Unclear - Not applicable |
| Was the condition measured in a standard, reliable way for all participants included in the case series? | - Yes - No - Unclear - Not applicable |
| Were valid methods used for identification of the condition for all participants included in the case series? | - Yes - No - Unclear - Not applicable |
| Did the case series have consecutive inclusion of participants? | - Yes - No - Unclear - Not applicable |
| Did the case series have complete inclusion of participants? | - Yes - No - Unclear - Not applicable |
| Was there clear reporting of the demographics of the participants in the study? | - Yes - No - Unclear - Not applicable |
| Was there clear reporting of clinical information of the participants? | - Yes - No - Unclear - Not applicable |
| Were the outcomes or follow up results of cases clearly reported? | - Yes - No - Unclear - Not applicable |
| Was there clear reporting of the presenting site(s)/clinic(s) demographic information? | - Yes - No - Unclear - Not applicable |
| Was statistical analysis appropriate? | - Yes - No - Unclear - Not applicable |

# References

Centers for Disease Control and Prevention. (2023). *Technical report: Highly pathogenic avian influenza A(H5N1) viruses.* Retrieved from <https://www.cdc.gov/flu/avianflu/spotlights/2022-2023/h5n1-technical-report_december.htm>

Chauhan, R. P., Dessie, Z. G., Noreddin, A., & El Zowalaty, M.,E. (2020). Systematic review of important viral diseases in africa in light of the 'one health' concept. *Pathogens (Basel, Switzerland), 9*(4) doi:10.3390/pathogens9040301

Chauhan, R. P., & Gordon, M. L. (2022). A systematic review of influenza A virus prevalence and transmission dynamics in backyard swine populations globally. *Porcine Health Management, 8*(1) doi:10.1186/s40813-022-00251-4

European Food, S. A., European Centre for Disease Prevention, and Control, European Union Reference Laboratory for, Avian Influenza, Adlhoch, C., Fusaro, A., Gonzales, J. L., . . . Kohnle, L. (2023). Avian influenza overview September–December 2023. *EFSA Journal, 21*(12), e8539. doi:10.2903/j.efsa.2023.8539

Food and Agricultural Organization of the United Nations. (2023). *Global avian influenza viruses with zoonotic potential situation update.* Rome, Italy: FAO. Retrieved from <https://www.fao.org/animal-health/situation-updates/global-aiv-with-zoonotic-potential/en>

Herzog, R., Álvarez-Pasquin, M. J., Díaz, C., Del Barrio, J. L., Estrada, J. M., & Gil, Á. (2013). Are healthcare workers’ intentions to vaccinate related to their knowledge, beliefs and attitudes? a systematic review. *BMC Public Health, 13*(1), 154. doi:10.1186/1471-2458-13-154

Kalonda, A., Phonera, M., Saasa, N., Kajihara, M., Sutcliffe, C. G., Sawa, H., . . . Simulundu, E. (2021). Influenza A and D viruses in non-human mammalian hosts in africa: A systematic review and meta-analysis. *Viruses, 13*(12) doi:10.3390/v13122411

Moola, S., Tufanaru, C., Aromataris, E., Sears, K., Sfetcu, R., Currie, M., . . . Mu, P. (2020). *JBI critical appraisal checklist for case reports.* Retrieved from <https://jbi.global/sites/default/files/2019-05/JBI_Critical_Appraisal-Checklist_for_Case_Reports2017_0.pdf.>

Munn, Z., Barker, T. H., Moola, S., Tufanaru, C., Stern, C., McArthur, A., . . . Aromataris, E. (2020). Methodological quality of case series studies: An introduction to the JBI critical appraisal tool. *JBI Evidence Synthesis, 18*(10), 2127-2133. doi:10.11124/JBISRIR-D-19-00099

Munn, Z., Moola, S., Lisy, K., Riitano, D., & Tufanaru, C. (2015). Methodological guidance for systematic reviews of observational epidemiological studies reporting prevalence and cumulative incidence data. *International Journal of Evidence-Based Healthcare, 13*(3), 147-153. doi:10.1097/XEB.0000000000000054

Pan American Health Organization, & World Health Organization. (2023). *Pan american health organization / world health organization. epidemiological update:
Outbreaks of avian influenza caused by influenza A(H5N1) in the region of the americas.* (). Washington, D.C: PAHO / WHO. Retrieved from <https://www.paho.org/en/documents/epidemiological-update-outbreaks-avian-influenza-caused-influenza-ah5n1-region-americas-0>

Public Health Agency of Canada. (2023). Human emerging respiratory pathogens bulletin: Issue 84, december 2023. Retrieved from <https://www.canada.ca/en/public-health/services/surveillance/human-emerging-respiratory-pathogens-bulletin/2023/december.html>

Tahmo, N. B., Wirsiy, F. S., Nnamdi, D., Tongo, M., Lawler, J. V., Broadhurst, M. J., . . . Brett-Major, D. (2023). An epidemiological synthesis of emerging and re-emerging zoonotic disease threats in cameroon, 2000-2022: A systematic review. *IJID Regions, 7*, 109. doi:10.1016/j.ijregi.2022.12.001

UK Health Security Agency. (2023). *Investigation into the risk to human health of avian influenza (influenza A H5N1) in england: Technical briefing 5.* London, England: UKHSA. Retrieved from <https://www.gov.uk/government/publications/avian-influenza-influenza-a-h5n1-technical-briefings/investigation-into-the-risk-to-human-health-of-avian-influenza-influenza-a-h5n1-in-england-technical-briefing-5>

Wells, G., O'Connell, D., Robertson, J., Welch, V. & Tugwell, P. (2021). The newcastle-ottawa scale (NOS) for assessing the quality of nonrandomised studies in meta-analyses. Retrieved from <https://www.ohri.ca/programs/clinical_epidemiology/nosgen.pdf>

World Health Organization. (2023a). *Avian influenza A (H5N1) - cambodia.* Geneva, Switzerland: WHO. Retrieved from <https://www.who.int/emergencies/disease-outbreak-news/item/2023-DON495>

World Health Organization. (2023b). *Avian influenza A (H5N1) - cambodia.* Geneva, Switzerland: WHO. Retrieved from <https://www.who.int/emergencies/disease-outbreak-news/item/2023-DON445>

World Health Organization. (2023c). *Avian influenza A (H5N1) - united states of america.* Geneva, Switzerland: WHO. Retrieved from <https://www.who.int/emergencies/disease-outbreak-news/item/2022-DON379>

World Health Organization. (2023d). *Influenza at the human-animal interface
summary and risk assessment, from 4 october to 1 november 2023.* Geneva, Switzerland: WHO. Retrieved from <https://cdn.who.int/media/docs/default-source/influenza/human-animal-interface-risk-assessments/influenza-at-the-human-animal-interface-summary-and-assessment--from-4-october-to-1-november-2023.pdf?sfvrsn=6c67e7df_2&download=true>

World Health Organization. (2024). WHO outbreak reports. Retrieved from <https://www.who.int/emergencies/disease-outbreak-news>
