# Supplementary material for: A Rapid Review Contrasting the Evidence on Avian Influenza A(H5Nx) Clades 2.3.4.4b and 2.3.2.1c in Humans
Source: Zoonoses Public Health. 2025 Aug 26;72(7):553–86. doi: 10.1111/zph.70006 (PMC12508786; doi:10.1111/zph.70006)
Supplement: Supplementary file 3 — Table S1: Detailed table of mutations associated with adaptation to mammals and/or humans within avian influenza isolates from humans infected with clades 2.3.2.1c and 2.3.4.4b. [file ZPH-72-553-s003.docx]

**Table S1: Detailed table of mutations associated with adaptation to mammals and/or humans within avian influenza A(H5Nx) isolates from humans infected with clades 2.3.2.1c (n= 5) and 2.3.4.4b (n=24)**

| **Reference** | **Clade** | **Human Isolate** | **Outcome** | **Details** |
| --- | --- | --- | --- | --- |
| (Takayama et al., 2016) | 2.3.2.1c A(H5N1) | A/Vietnam/14011801/2014 | Receptor specificity | Virus was shown to predict binding specificity to an avian α2,3 sialic acid receptor. |
|  |  |  | Increased adaptation, virulence, infectivity, transmissibility in mammalian hosts | No amino acid substitutions associated with increased adaptation, virulence, infectivity, or transmissibility in mammalian hosts, including the E627K and D701N mutations in polymerase basic protein 2. |
| (Pabbaraju et al., 2014) | 2.3.2.1c A(H5N1) | A/Alberta/01/2014 | Receptor specificity | The sequence of the 220-loop receptor binding site (RBS) contained the typical avian amino acids, Q222/G224, predictive of a preference for the avian α2,3 rather than the human α2,6 sialic acid host cell receptor.  Mutations D94N, S133A, S155N, and T156A found. D94N decreased binding to avian α2,3 sialic acid and increased it to human α2,6 sialic acid in a pseudotyping assay. When together, S155N and T156A also increased binding to α2,6 sialic acid (assayed with resialated erythrocytes). |
|  |  |  | Increased binding to human erythrocytes | G221R substitution was detected which has been shown to slightly increase binding to human erythrocytes. |
|  |  |  | Airborne transmission | Mutation T156A. T156A is consistently found in ferret-adapted mutants capable of airborne transmission. |
|  |  |  | Improved viral replication | Mutation T156A. T156A abrogates a N-glycosylation site and, when together with S223N (not found in A/Alberta/01/2014), may improve virus replication in the upper respiratory tract of ferrets. |
|  |  |  | Increased virulence in mice | Mutations N30D and T215A found in the M1 gene and are associated with increased virulence in mice.  Several NS1 mutations reported to increase virulence in mice were present: P42S, D87E, L98F, and I101M; a 4-bp deletion from nt 80–84, along with the D92E shift; and the PDZ ligand domain (ESEV) at the C terminus.  -PB2 substitutions (Amino acid changes L89V, G309D, T339K, R477G, I495V, and K627E and a change to Met at the predicted position A676T) in conjunction with changes in the M1 and HA proteins (only some of which were identified in this isolate) have been described to enhance polymerase activity and virulence in mice. |
|  |  |  | No mammalian adaptation | The PB2 sequence showed the presence of E627, establishing the lack of a well-known mammalian adaptation motif. |
|  |  |  | Enhanced polymerase activity in mammalian cells and mice | The PB1 protein showed the P598L mutation reported to enhance polymerase activity in mammalian cells and mice  Of the polymerase mutations hypothesized to increase the RNA polymerase activity of HPAI A(H5N1) viruses, namely P149S, R226H, K357I, and T515S, only two, 149S and 357T, were present in the A/Alberta/01/2014 isolate.  Two polymerase mutations hypothesized to increase RNA polymerase activity were found: 149S and 357T. |
|  |  |  | No increase in replication efficiency, virulence, transmission | Mutations in the nucleoprotein gene reported to enhance replication efficiency, virulence, and transmission were absent in the isolate. |
| (Suttie et al., 2019) | 2.3.2.1c A(H5N1) | A/Cambodia/Y0219302/2014 | Viral fitness | Two amino acid changes were found in the human isolate (I151L and F364Y) compared to two highly similar (99.5%) poultry isolates but neither mutation has been associated with a significant increase in viral fitness.  HA protein gene mutations found that are associated with increased viral fitness: D101N, S137A, S159N, T160A, K193R, N158D |
|  |  |  | Increase in viral pathogenicity | Multibasic cleavage site found in HA protein can increase viral pathogenicity. |
|  |  |  | Increase in specificity for α2,6 human-type receptors, increased transmission in guinea pigs | Protein HA, Mutation |
|  |  |  | Increased replication in avian cells and virulence in chickens | Protein NP, mutation M105V and A184K found |
|  |  |  | Receptor specificity | The well-known Q222L and G224S substitutions associated with increased binding to α2,6 receptors and decreased α2,3 binding were not observed  HA sequences also contained mutations associated with an increase in sialic acid receptor binding to α2,6 human-type receptors, including: D94N, S133A, S155N, T156A, T188 and K189R (although numerous substitutions were also identified that are associated with a binding preference for α2,3 receptors) |
| (Pardo-Roa et al., 2023) | 2.3.4.4b A(H5N1) | A/Chile/25945/2023 | Adaptation to mammalian hosts | D701N and Q591K substitutions in PB2 were observed in the human case. D701N has been shown to enhance viral replication and pathogenicity in mammalian hosts, including humans and Q591K is implicated in increased replication and transmission of the virus in mammals. |
| (Zhang et al., 2022) | 2.3.4.4b A(H5N6) | A/Yangzhou/125/2022 | Receptor specificity | The amino acid motif at residues 226–228 (H3 numbering) in HA was Gln-Ser-Gly, suggesting its preference for an avian-like receptor. However, the receptor-binding site Thr160Ala mutation might enhance affinity with the human-type receptor. |
|  |  |  | Enhanced virulence in mice | A deletion of 12 amino acids at positions 59–70 in the NA stalk and5 amino acids at positions 80–84 in the NS1 associated with increased virulence in mice were observed.  Many mutations in the internal genes associated with enhanced virulence in mice, including Leu89Val, Gly309Asp, Thr339Lys, Arg477Gly, Ile495Val, Ala676Thr in PB2, Ser622Gly in PB1,Ser515Thr in PA, Asn30Asp, Thr215Ala, Ile43-Met in M1, Pro42Ser, Val149Ala,Cys138Leu, Leu103Phe, Ile106Met in NS1 were revealed. |
| (Liu et al., 2022) | 2.3.4.4b A(H5N6) | A/Sichuan/06681/2021  A/Hunan/09285/2021  A/Hunan/10117/2021  A/Hunan/09911/2021  A/GX/guilin/11151/2021  A/Hangzhou/01/2021 | Receptor specificity | All viruses contained more than one of the seven HA amino acid mutations (94N (n=0/6), 133A (n=6/6), 154D (NR), 155N (NR), 156A (NR), 188I (n=6/6) and 189R (n=0/6)), which could increase virus binding to α2, 6-linked sialic acid receptors. |
|  |  |  | Virulence, transmission, replication efficiency in mammals | Most viruses contained the NS1 103F (n=5/6) and NS1 106M (n=6/6) mutations associated with virulence, transmission, replication efficiency and adaptation in mammals. |
| (Zhu et al., 2022) | 2.3.4.4b A(H5N6) | 19 isolates  A/Guangxi/04901/2021  A/Guangxi/04900/2021  A/GX-guilin/11151/2021  A/Guangxi/10287/2021  A/Guangxi/10285/2021  A/Hangzhou/01/2021  A/Hunan/09911/2021  A/Hunan/11318/2021  A/Hunan/10117/2021  A/Hunan/09285/2021  A/Guangdong/12903/2021  A/Sichuan-Luzhou/LZ20211439-Q1/2021  A/Sichuan/ZGQ2021002/2021 A/Sichuan/06689/2021  A/Sichuan/06681/2021  A/Chongqing/03/2021  A/Chongqing/02/2021  A/Sichuan/07348/2021  A/Fujian-Sanyuan/21099/2017 | Receptor specificity | Substitution Q226L (H3 numbering) in HA protein, which had previously been reportedly associated with a switch in receptor specificity from avian-type (α2–3Gal) to human-type (α2–6Gal), was detected in 2/19 viruses. Could increase the viral affinity for human cells, indicating the viral adaptation process from birds to humans.  Substitution S227R in HA protein, which could also alter receptor specificity, was detected in 19/19 2.3.4.4b viruses.  Another receptor-changing substitution T192I, was newly detected in clade 2.3.4.4b viruses from 2021 cases (n=18/18) and not detected in 2017 cases (n=0/1). Could increase the viral affinity for human cells, indicating the viral adaptation process from birds to humans. |
|  |  |  | Increased polymerase activity or enhanced virulence in mice | Several mammalian-adapted mutations have occurred in PB2 protein of H5N6 viruses. Substitutions E627K and D701N in PB2 protein (E627K = 2/19, D701N = 3/19 were associated with increased polymerase activities or enhanced virulence in mice. |
| (Gu et al., 2022) | 2.3.4.4b A(H5N6) | A/Hunan/09911/2021  A/Hunan/10117/2021  A/Hangzhou/01/2021  A/Hunan/09285/2021  A/GX_guilin/11151/2021  A/Sichuan/06689/2021  A/Sichuan/06681/2021 | Increased replication and virulence in mammals | The E627K or D701N substitutions in PB2 that increase virulence in mammals were not detected in these H5N6 viruses; however, the following amino acid residues linked to increased replication and virulence in mammals were detected: 225G in HA, 89V, 292V, 309D, 389R, and 598T in PB2, 622G in PB1, 30D, 43M, and 215A in M1, and 42S and 106M in NS1. |
|  |  |  | Receptor specificity | Although the Q226L or G228S amino acid substitutions were not detected in the HA, all viruses have the amino acids 137A, 158N, 160A, and 186N in their HA, and 7 human isolates have the amino acid 192I in their HA (H3 numbering), which have been reported to increase the affinity of H5 influenza virus for human-type receptors. |
| (Li et al., 2022) | 2.3.4.4b A(H5N6) | A/GX_guilin/11151/2021 | Adaptation | Isolate contained two substitutions in the PA (R635 K) and NP (E434 K) proteins compared with those from the slaughtered poultry, suggesting that the variant was adapting to the host after infection. |
| (Briggs & Kapczynski, 2023) | 2.3.4.4b A(H5Nx) | Not provided, there are 5.3k sequences analysed from many species. | Mammalian host adaptation | Two isolates contained the PB2 E627K mutation. Analysis shows that this mutation is associated with adaptation to human hosts and infection in a wider range of species than would be expected and has become more prevalent in 2.3.4.4b isolates since 2021 (48/53). |
| (Ding, Li, Li, & Qu, 2022) | 2.3.4.4b A(H5N8) | A/Astrakhan/3212/2020 | Receptor specificity | -Human-to-human transmission appears to be limited, since the virus had QRG but not LRS residues at the receptor-binding site.  -On a molecular basis, the human virus lacks Q226L and G228S mutations in the receptor-binding site. This suggests that the new virus retains weak binding activity to α–2,6 sialic acid receptor, which is highly expressed in the human airway epithelial. -The virus has also 137A, 158N, 160A, and 186N residues in its HA protein, which have been reported to increase the affinity to α–2,6 sialic acids, a prerequisite for efficient human transmission. |
| (Oliver et al., 2022) | 2.3.4.4b A(H5N1) | A/England/215201407/2021 |  | The spill-over infection to the human contact did not lead to any detected genetic changes in the virus that might be associated with increased zoonotic risk.  Analysis confirmed that the H5N1 in human respiratory material was identical in all segments – at the consensus level – to the avian sequence, apart from four nucleotide (nt) mutations: three synonymous mutations, two in the polymerase basic protein (PB) 2 gene at nt positions 75 and 220, and one in PB1 gene at nt position 1,481. One coding change was noted in polymerase acidic protein (PA) gene arising from a further non-synonymous mutation at nt position 485, resulting in a conservative change from asparagine (N) in the duck sequence to threonine (T) at amino acid position 162 in the human PA sequence. In all other UK H5N1 avian genomes from 2021, there is a T at position 162. |
| (Xiao et al., 2021) | 2.3.4.4b A(H5N6) | A/Sichuan/06681/2021  A/Sichuan-Bazhong/1/2021 | Receptor specificity | Host-specific related sites, such as receptor binding Q226L of HA fragment and E627K of PB2 fragment were not found, which indicated that both strains still possess features of avian origin. |
|  |  |  | Increased virulence in chickens | The cleavage site of HA protein possessed a multiple basic amino acids motif (LREKRRKR↓G), which indicated high pathogenicity to chickens. |
|  |  |  | Enhanced viral replication in mammalian cells in vitro | Gene - PB2, Amino acid position - T271A, mutation T1648. |
|  |  |  | Increased pathogenicity in mice | Gene PB2, Amino acid position Q591K, mutation Q1693. |
|  |  |  | Increased virulence in mammals | Gene - PB2, Amino acid position - E627K, mutation E1673. |
|  |  |  | Altered virulence in mice | Gene PB2, Amino acid position - D701N, mutation D1689. |
|  |  |  | Promotes viral replication in mice | Gene NS1, Amino Acid position P212S, mutation P1681. |
|  |  |  | Increase virulence and/or cytokine resistance | Gene NS1, Amino acid position D92E, mutation D355. |
| (Bi et al., 2021) | 2.3.4.4b A(H5N6) | A/GX-guilin/11151/2021 | Viral replication | K389R, V598T/I mutations of PB2 gene, and N409S mutation of PA gene, which could increase virus replicative ability in mammals, was observed. |
|  |  |  | Receptor specificity | The receptor-binding site at the 222–224 motif was QRG suggesting that this virus preferred binding to avian-like receptors (α 2,3 SA). However, S133A, D155N, T156A, and T188I mutations in the GX11151 HA gene increased virus binding to human-like receptors (α2-6 SA). |
|  |  |  | Mammalian virulence/pathogenicity | The HA cleavage site possessed a multiple basic amino acids motif, indicating potentially high pathogenicity in chickens. A263T mutation in HA gene was detected suggesting that the virulence was enhanced. Some mutations increasing virulence in mice were detected, such as N30D, T139A, and T215A of M1 gene and P42S and 80–84 deletion of NS1 gene. |

## References

References

Bi, F., Jiang, L., Huang, L., Wei, J., Pan, X., Ju, Y., . . . Wang, J. (2021). Genetic characterization of two human cases infected with the avian influenza A (H5N6) viruses - guangxi zhuang autonomous region, china, 2021. *China CDC Weekly, 3*(44), 923-928. doi:10.46234/ccdcw2021.199

Briggs, K., & Kapczynski, D. R. (2023). Comparative analysis of PB2 residue 627E/K/V in H5 subtypes of avian influenza viruses isolated from birds and mammals. *Frontiers in Veterinary Science, 10* doi:10.3389/fvets.2023.1250952

Ding, L., Li, J., Li, X., & Qu, B. (2022). Evolutionary and mutational characterization of the first H5N8 subtype influenza A virus in humans. *Pathogens (Basel, Switzerland), 11*(6) doi:10.3390/pathogens11060666

Gu, W., Shi, J., Cui, P., Yan, C., Zhang, Y., Wang, C., . . . Chen, H. (2022). Novel H5N6 reassortants bearing the clade 2.3.4.4b HA gene of H5N8 virus have been detected in poultry and caused multiple human infections in china. *Emerging Microbes & Infections, 11*(1), 1174-1185. doi:10.1080/22221751.2022.2063076

Li, J., Fang, Y., Qiu, X., Yu, X., Cheng, S., Li, N., . . . Wang, H. (2022). Human infection with avian-origin H5N6 influenza a virus after exposure to slaughtered poultry. *Emerging Microbes & Infections, 11*(1), 807-810. doi:10.1080/22221751.2022.2048971

Liu, H., Wu, C., Pang, Z., Zhao, R., Liao, M., & Sun, H. (2022). Phylogenetic and phylogeographic analysis of the highly pathogenic H5N6 avian influenza virus in china. *Viruses, 14*(8), 1752. doi: 10.3390/v14081752. doi:10.3390/v14081752

Oliver, I., Roberts, J., Brown, C. S., Byrne, A. M., Mellon, D., Hansen, R. D. E., . . . Zambon, M. (2022). A case of avian influenza A(H5N1) in england, january 2022. *Euro Surveillance : Bulletin Europeen Sur Les Maladies Transmissibles = European Communicable Disease Bulletin, 27*(5) doi:10.2807/1560-7917.es.2022.27.5.2200061

Pabbaraju, K., Tellier, R., Wong, S., Li, Y., Bastien, N., Tang, J. W., . . . Tipples, G. A. (2014). Full-genome analysis of avian influenza A(H5N1) virus from a human, north america, 2013. *Emerging Infectious Diseases, 20*(5), 887-891. doi:10.3201/eid2005.140164

Pardo-Roa, C., Nelson, M. I., Ariyama, N., Aguayo, C., Almonacid, L. I., Munoz, G., . . . Neira, V. (2023). Cross-species transmission and PB2 mammalian adaptations of highly pathogenic avian influenza A/H5N1 viruses in chile. *bioRxiv : The Preprint Server for Biology,* doi:10.1101/2023.06.30.547205

Suttie, A., Tok, S., Yann, S., Keo, P., Horm, S. V., Roe, M., . . . Horwood, P. F. (2019). Diversity of A(H5N1) clade 2.3.2.1c avian influenza viruses with evidence of reassortment in cambodia, 2014-2016. *PloS One, 14*(12), e0226108. doi:10.1371/journal.pone.0226108

Takayama, I., Hieu, N. T., Shirakura, M., Nakauchi, M., Fujisaki, S., Takahashi, H., . . . Kageyama, T. (2016). Novel reassortant avian influenza A(H5N1) virus in human, southern vietnam, 2014. *Emerging Infectious Diseases, 22*(3), 557-559. doi:10.3201/eid2203.151360

Xiao, C., Xu, J., Lan, Y., Huang, Z., Zhou, L., Guo, Y., . . . Yang, H. (2021). Five independent cases of human infection with avian influenza H5N6 - sichuan province, china, 2021. *China CDC Weekly, 3*(36), 751-756. doi:10.46234/ccdcw2021.187

Zhang, L., Liu, K., Su, Q., Chen, X., Wang, X., Li, Q., . . . Zhang, P. (2022). Clinical features of the first critical case of acute encephalitis caused by the avian influenza A (H5N6) virus. *Emerging Microbes & Infections, 11*(1), 2437-2446. doi:10.1080/22221751.2022.2122584

Zhu, W., Li, X., Dong, J., Bo, H., Liu, J., Yang, J., . . . Wang, D. (2022). Epidemiologic, clinical, and genetic characteristics of human infections with influenza A(H5N6) viruses, china. *Emerging Infectious Diseases, 28*(7), 1332-1344. doi:10.3201/eid2807.212482
