# Supplementary material for: A Rapid Review Contrasting the Evidence on Avian Influenza A(H5Nx) Clades 2.3.4.4b and 2.3.2.1c in Humans
Source: Zoonoses Public Health. 2025 Aug 26;72(7):553–86. doi: 10.1111/zph.70006 (PMC12508786; doi:10.1111/zph.70006)
Supplement: Supplementary file 4 — Table S2: Pathogenicity and virulence of avian influenza isolates from birds and mammals infected with 2.3.2.1c and 2.3.4.4b in human cell lines. [file ZPH-72-553-s001.docx]

**Table S2: Pathogenicity and virulence of avian influenza A(H5Nx) isolates from birds and mammals infected with clades 2.3.2.1c (n=1) and 2.3.4.4b (n=4) in human cell lines**

| **Reference** | **Clade and isolate(s)** | **Cell line** | **Outcome** |
| --- | --- | --- | --- |
| (Yang et al., 2019) | 2.3.2.1c A(H5N1)  Three isolates:  Chickens - A/chicken/Gansu/ XG2/2012  A/chicken/Qinghai/QH3/2015  Environment -  A/environment/Qinghai/  1/2013 | Human A549 cells (epithelial cell line) and Madin-Darby canine kidney (MDCK) (canine cell line) | **Replication**  In human A549 cells, the viral replication of the two strains had a similar trend with that in MDCK cells from 12-72 hours post-infection, however titers were lower at the initial timepoint.  The results of viral replication kinetics indicated that the viruses were capable of replicating well in mammalian (canine) and human cells. |
| (Kobasa et al., 2023)  *Preprint* | 2.3.4.4b A(H5N1)  Four isolates:  Red tailed hawk - A/Red Tailed Hawk/ON/FAV-0473-4/2022 (RT.Hawk/ON/22)  Redfoxes -A/Redfox/PEI/FAV-0544/2022 (Redfox/PEI/22)  A/Redfox/ON/FAV-0301-5/2022 84 (Redfox/ON/22)  Turkey - A/Turkey/ON/  FAV-0162-144/2022 (Turkey/ON/22) | Human epithelial cells (primary nasal, bronchial/tracheal, and small airway cells) | **Replication**  The red-tailed hawk isolate replicated most rapidly of the four viruses in all three epithelial cell types with peak titers in all cell lines between 3-5 days post-infection.  The turkey isolate replicated poorly in all cultures, while results for both red fox viruses were variable, depending on the cell type.  Redfox/ON/22 replicated to modest titers on bronchial and small airway cells but poorly in nasal cells.  Redfox/PEI/222 attained a higher titer than  RT.Hawk/On/22 in small airway cells, but replicated slower, peaking between days 10-14, yet replicated poorly in bronchial and with delayed kinetics in nasal cells.  RT.Hawk/ON/22 replicated to high virus titers in human nasal epithelial cells.  Data demonstrates the significant replicative ability and transmission potential of RT.Hawk/ON/22 as this virus replicates efficiently in cells of the upper respiratory tract, which could result in higher shedding of virus.  **Virulence**  Data from animal models of disease in white-footed mice and ferrets suggest the most virulent isolate is RT.Hawk/ON/22 > Redfox/ON/22> Turkey/ON/22> Redfox/PEI/22. Data not directly relevant to this review.  **Transmission**  Viral titers in oral, nasal or fecal samples suggested the highest risk of transmission from direct contact in a ferret model was from RT.Hawk/ON/22 > Redfox/ON/22> Turkey/ON/22> Redfox/PEI/22.  From airborne virus exposure to RT.Hawk/ON/22 ferrets had detectable RNA in respiratory samples, but the experiment may have ended prior to clinical disease development. Data shows RT.Hawk/ON/22 was well adapted to transmit between mammals in the animal models. |
| (Bui et al., 2021) | 2.3.4.4b A(H5N6/H5N8)  Three isolates:  Black-faced spoonbill -H5N6 A/spoonbill/HK/1718259/2017  Chicken - H5N8 A/chicken/Egypt/F1366A/2017  Grey-headed gull -H5N8 A/grey-headed gull/Uganda/ 200144/2017 | Human airway organoids and primary human alveolar epithelial cells | **Replication**  All isolates demonstrated productive replication in human airway organoids and alveolar epithelial cells (multiplicities of infection 0.01); by 72 h after infection, mean peak titers were 3.7–5.1 log TCID50/mL for human airway organoids and 4.6–7.0 log TCID50/mL for alveolar epithelial cells.  Replication of bird AIVs was less efficient than that of human isolates (A(H5N1) 2.3.2.1b, A(H5N6) 2.3.4.4, A(H1N1) clade 0, pH1N1) in human airway organoids and in alveolar epithelial cells (except pH1N1 which did not replicate efficiently). Not all differences were statistically significant.  Suggests lower zoonotic potential and transmissibility of these avian isolates in humans compared to the human isolates studied. Thus, the risk for human-to-human transmission of 2.3.4.4b bird isolates from 2016-2018 is low, but zoonotic transmission is possible.  **Cellular Tropism**  According to immunohistochemistry double staining, the avian isolate (A(H5N6) - spoonbill infected acetyl-α-tubulin–positive ciliated cells, SCGB1A1–positive/CC10–positive secretory club cells, MUC5AC–positive secretory goblet cells, and p63-α–positive basal cells, similar to human isolates (A(H5N1) 2.3.2.1b, A(H5N6) 2.3.4.4, A(H1N1) clade 0, pH1N1)  **Proinflammatory Cytokine and Chemokine Induction**  Compared with human isolates (A(H5N1) 2.3.2.1b, A(H5N6) 2.3.4.4, A(H1N1) clade 0, pH1N1), the clade 2.3.4.4b bird isolates induced fewer proinflammatory cytokines and chemokines  At 24 h after infection, human isolates tended to induce higher mRNA levels of IFN-β, IFN-λ1, CCL5, CXCL10, TNFα, IL-6, ISG15, and MX1 than most clade 2.3.4.4b bird isolates in human airway organoids and in alveolar epithelial cells; statistical significance for IFN-λ1, CXCL10, and MX1 in human airway organoids, and IFN-β, IFN-λ1, CCL5, CXCL10, ISG15, and MX1 in alveolar epithelial cells.  No statistically significant differences in the mRNA levels of IFN-β, IFN-λ1, CCL5, CXCL10, TNFα, IL-6, ISG15, and MX1 between avian isolates 2.3.4.4b in human airway organoids. Similarly, there was only a few differences between their IFN-β, TNFα, ISG15, and MX1 mRNA levels in alveolar epithelial cells. |
| (Blaurock et al., 2021) | 2.3.4.4b A(H5N8)  One isolate:  Tufted duck - A/tufted duck/Germany /8444/2016 (NS217) | Human A549 cells (epithelial cell line), human embryonic kidney 293T (HEK293T) cells, MDCK-II cells (canine) | This study examined NS1 sequences from AIV sequences available in GISAID. Overall observation was that human AIV isolates and isolates from 2.3.4.4b (2016-2020) tended to have short NS1 sequences (NS217-NS219) compared to bird AIVs and clade 2.3.4.4a (NS230-NS237).  **Replication**  For 2.3.4.4b, the original isolate NS217 was most efficient at cell-to cell spread. Altered NS1 lengths to NS230 or NS237 resulted in significantly smaller plaque size, p<0.001.  Replication of the original NS217 and altered NS230 or NS237 were similar in avian DF1, human A549, canine MDCK and MDCK-II cell lines.  **Interferon Induction**  The alteration of 2.3.4.4b NS217 to NS230 amino acids significantly reduced its ability to block both IFN-α and IFN-β mRNA expression in avian DF-1 cells.  In human A549 cells, viruses carrying B_NS217 were most efficient at blocking the IFN-β and IFN-α response and that extension to NS230 and NS237 gradually reduced this efficiency.  **Apoptosis Induction**  Infected A549 cells NS217, NS230 or NS237 had detectable caspase-3 (signalling apoptosis) at 24 hours. The signal was significantly weaker for NS217 indicating the shorter NS1 original variant induce apoptosis less extensively during infection, and that this is the case in both infected cells and in uninfected bystander cells. |
| (Zhang et al., 2023) | 2.3.4.4b A(H5N8)  One isolate: A/goose/Hebei/HG12/2021 | Human A549 and MDCK cells | **Replication**  This isolate replicated more effectively in A549 ​cells compared to MDCK cells, suggesting that it has potential to infect humans and mammals. |

## References

Blaurock, C., Blohm, U., Luttermann, C., Holzerland, J., Scheibner, D., Schäfer, A., . . . Abdelwhab, E. M. (2021). The C-terminus of non-structural protein 1 (NS1) in H5N8 clade 2.3.4.4 avian influenza virus affects virus fitness in human cells and virulence in mice. *Emerging Microbes & Infections, 10*(1), 1760-1776. doi:10.1080/22221751.2021.1971568

Bui, C. H. T., Kuok, D. I. T., Yeung, H. W., Ng, K., Chu, D. K. W., Webby, R. J., . . . Chan, M. C. W. (2021). Risk assessment for highly pathogenic avian influenza A(H5N6/H5N8) clade 2.3.4.4 viruses. *Emerging Infectious Diseases, 27*(10), 2619-2627. doi:10.3201/eid2710.210297

Kobasa, D., Warner, B., Alkie, T., Vendramelli, R., Moffat, E., Tailor, N., . . . Berhane, Y. (2023). Transmission of lethal H5N1 clade 2.3.4.4b avian influenza in ferrets. *Research Square*. doi:10.21203/rs.3.rs-2842567/v1

Yang, J., Wang, Z., Du, Y., Jia, Y., Wang, L., Xu, S., & Zhu, Q. (2019). Clade 2.3.2.1 H5N1 avian influenza viruses circulate at the interface of migratory and domestic birds around qinghai lake in china. *Veterinary Microbiology, 235*, 234-242. doi:10.1016/j.vetmic.2019.07.009

Zhang, C., Wang, Z., Cui, H., Chen, L., Zhang, C., Chen, Z., . . . Guo, Z. (2023). Emergence of H5N8 avian influenza virus in domestic geese in a wild bird habitat, yishui lake, north central china. *Virologica Sinica, 38*(1), 157-161. doi:10.1016/j.virs.2022.10.002
